# Validation of the Brief Index of Sexual Functioning for women and men (BISF-W and BISF-M) in an Italian sample

**DOI:** 10.3389/fpsyg.2024.1474288

**Published:** 2024-11-06

**Authors:** Marta Panzeri, Lucia Ronconi, Lilybeth Fontanesi

**Affiliations:** ^1^Department of Developmental Psychology and Socialization, School of Psychology, University of Padua, Padua, Italy; ^2^Computer and Statistical Services, Multifunctional Pole of Psychology, University of Padua, Padova, Italy; ^3^Department of Psychology, G. D'Annunzio University of Chieti and Pescara, Chieti, Italy

**Keywords:** BISF, sexual functioning, dyadic sexuality, solitary sexuality, gender differences, age differences

## Abstract

**Introduction:**

The Brief Index of Sexual Functioning for Women (BISF-W) is an international 4-factors tool assessing qualitative and quantitative aspects of sexual experiences in women, both in clinical and experimental settings. The present research aims at validating an Italian version of the BISF-W, to develop a BISF version for men (BISF-M) to fill the gap in the existing sexual function evaluation tools in Italy and to analyze gender and age groups differences in the BISF factors.

**Methods:**

The research included 6,355 women, aged from 18 to 65 (*M* = 34.94, *SD* = 10.52) and 2,207 men, aged from 18 to 80 (*M* = 38.25, SD = 13.67), who completed the BISF-W and BISF-M. The Quality of Marriage Index (QMI) was administrated to both samples for testing divergent validity, while Female Sexual Function Index (FSFI) and the International Index of Erectile Function (IIEF) were administered for testing convergent validity. Correlation analysis, MANOVA between gender and age and Confirmatory Factor Analysis were conducted.

**Results:**

The CFAs confirmed that the proposed 4-factor model (Dyadic, Solitaire and Anal Sexuality, and Sexual Difficulties) is suitable both for the BISF-W and the BISF-M, demonstrated strong psychometric properties for assessing sexual functioning in both genders, with dyadic sexuality being the most important factor. MANOVA analysis showed significative differences in the factors’ scores, according to gender and age.

**Discussion:**

The BISF-W and the BISF-M are promising tools to address sexual functioning in individuals and couples, both in clinical and non-clinical settings. Gender and age differences are discussed regarding the potential use of BISF in the therapeutic context.

## 1 Introduction

The Brief Index of Sexual Functioning for Women (BISF-W) is a self-report questionnaire developed in 1994 by Taylor and colleagues. It comprises 22 questions, totaling 64 items, that assesses female sexual functioning over the past 30 days. Two questions were posed in a binary format, 15 were constructed with a single item and offered four to eight options, and eight questions encompassed several items (ranging from five to eight) and employed a Likert scale with five to seven points, such as “Not at all,” “Once,” “2 or 3 times,” “Once a week,” “2 or 3 times per week,” “Once a day,” and “More than once a day” (see S1.1 in Supporting Information). Responses are primarily measured on a Likert scale, which varies depending on the question. It covers a very wild spectrum of (female) sexuality, ranging from the frequency of different sexual activities such as desire, kissing, solitary masturbation, mutual masturbation, oral, vaginal and anal sex, to measuring the level of arousal provoked by the same activities, the frequency of orgasm provoked by them, sexual satisfaction, to sexual problems (this is the only section in which there is an item relating only to female sexuality). This feature leads us to choose this tool, since it seems more complete than others that can have a more linear structure, such as the Arizona Sexual Experience Scale (ASEX, Gelenberg et al., 1997) which is made up of only 5 elements, but in our opinion are not sufficient to study human sexuality in depth. The ASEX focuses on 5 domains as sexual drive, arousal, lubrication/erection, ability to reach orgasm and satisfaction, but it lacks depth in areas like sexual pain, emotional intimacy, and relational aspects of sexual function and is not gender-sensitive as it is not tailored to the complexity of the female sexual experience (Wiegel et al., 2005; Zemishlany and Weizman, 2008).

The psychometric properties of the original version of the questionnaire (Taylor et al., 1994) were validated through an exploratory factor analysis (EFA) based on principal component analysis with varimax rotation, performed on all questions except the first two and the last two, which investigate, respectively, the presence/absence of a partner and the type of partners (permanent or casual) and sexual orientation (heterosexual, gay/lesbian or bisexual). For questions consisting of multiple items, the mean was presumably used. From this initial analysis, three factors were identified: Sexual Interest/Desire (for a total of 5 questions, 12 items, such as Question 8: “During the past month, who has usually initiated sexual activity?”), which measures interest or desire for sexual activity; Sexual Activity (for a total of 9 questions, 36 items, such as Question 4: “Using the scale to the right, indicate how frequently you have felt a desire to engage in the following activities during the past month? –Kissing; −Masturbation alone; −Mutual masturbation; –Petting and foreplay; –Oral sex; –Vaginal penetration or intercourse; −Anal sex”), which assesses sexual activity or frequency; and Sexual Satisfaction (for a total of 6 questions, 10 items, such as Question 18: “Overall, how satisfied have you been with your sexual relationship with your partner?”), which assesses pleasure, communication, and satisfaction with the sexual relationship. Five questions (5 items) were bifactorial (for example Question 6: “Overall, during the past month, how frequently have you become anxious or inhibited during sexual activity with a partner?”). Overall, these dimensions accounted for 51.2% of the total variance. The questionnaire’s reliability was satisfactory, except for the Sexual Interest/Desire factor, which had Cronbach’s alphas of 0.39, 0.83, and 0.74, respectively. The principal issue with this analysis are the five questions (5 items) that were bifactorial and the three saturated with no factor.

In a second step, Mazer et al. (2000) developed a quantitative scoring algorithm from a conceptual basis to provide an overall sexual function score (composite score or c-score) and 7 scores related to 7 dimensions representing the main parameters of female sexual function: Thoughts/Desire, Arousal, Receptivity/Initiation, Pleasure/Orgasm, Relationship Satisfaction, Problems affecting sexual function, and Frequency of sexual activity. The Cronbach’s alphas for the Arousal (*α* = 0.39), Receptivity/Initiation (*α* = 0.45), and Frequency of sexual activity (*α* = 0.08) subscales appear unsatisfactory, while those for the Thoughts/Desire (*α* = 0.72), Pleasure/Orgasm (*α* = 0.72), and Problems affecting sexual functioning (*α* = 0.61) subscales were satisfactory. The alpha for the frequency of sexual activity dimension was not calculated. The dimensions of Thoughts/Desire, Arousal, and Pleasure/Orgasm align with Kaplan’s triphasic model of the sexual response cycle (Derogatis, 1998) which has been proposed as a framework for assessing female sexual dysfunction (Derogatis, 1997). The dimension Frequency of sexual activity serves as an index for the amount and diversity of sexual activity during the sexual response cycle. On the other hand, the dimension Receptivity/Initiation can be seen as a behavioral manifestation of sexual desire. The dimensions of Relationship satisfaction and Problems affecting sexual function are important indicators of the emotional context in which sexual activity occurs, as well as potential issues that can negatively impact sexual functioning. The primary issue with this algorithm is the absence of a confirmatory factor analysis (CFA) to validate the model. Additionally, the reliability of numerous dimensions is low.

In Han et al. (2014), based on the limitation of previous scores (Mazer et al., 2000; Taylor et al., 1994) developed a Chinese version of the BISF-W. They administrated it to 93 healthy women and 113 women with recurrent depression. Based on an EFA run with principal component analysis and varimax rotation, they identified four factors, different from those identified by Taylor et al. (1994) and Mazer et al. (2000). Factor 1 pertains to sexual interaction, including expectations, initiation, response, communication, and enjoyment. Factor 2 represents the physical aspects of sexual activity. Factor 3 illustrates the adverse effects of sexual activity, such as sexual dysfunctions and factors that impact sexual function. Factor 4 comprises solely of two components: sexual thought and sexual attitude, which demonstrate a subjective viewpoint on sex. The Cronbach’s alpha values were satisfactory for the first three factors, ranging from 0.86 to 0.74, but inadequate for the fourth factor, which had a value of 0.36. To our knowledge in other languages there are only a linguistic validation in French (Baudelot-Berrogain et al., 2006) that used Mazer’ 7 dimensions (Mazer et al., 2000) and assessed the influence of organic variables on female sexuality without any statistical analysis and a Czech translation (Heřmánková et al., 2021) that was used to asses sexuality in patients affected by rheumatic diseases.

For a preliminary validation of the Italian version of the BISF-W, it was first translated into Italian using the back translation technique (Panzeri and Optale, 2006). An exploratory factor analysis (EFA) was conducted on a sample of 1,051 Italian women.

Principal component analysis and Oblimin rotation were used to analyze all items, assuming that factors were not orthogonal to each other (Panzeri et al., 2009). The study found a four-factor structure that explained 48.67% of the variance, with satisfactory reliability values. This is in contrast to the original version (Taylor et al., 1994), which identified only three factors, and the new scoring algorithm version (Mazer et al., 2000), which identified seven dimensions. Questions 8 (“Who has usually initiated sexual activity?”), 15 [“Indicate the frequency with which the following factors have influenced your level of sexual activity: —My own health problems (for example, infection, illness); —My partner’s health problems; —Conflict in the relationship; —Lack of privacy; —Other (please specify):___”], and 16 (“How satisfied are you with the overall appearance of your body?”) were removed as they did not contribute to any factors. The initial factor, labeled Dyadic Sexuality, encompasses desires, frequency, arousal, and orgasm achieved during shared activities (such as kissing, mutual foreplay and fondling, mutual stimulation, oral intercourse, and coitus). The second factor, named Solitary Sexuality, includes desires, frequency, arousal, and achieved orgasm in activities that are performed alone, such as masturbation, fantasies, or erotic dreams. The third factor, named Sexual Difficulties, refers to the level of satisfaction with one’s sexual relationship with their partner and any issues that may impact sexual activity, such as physical or psychological problems. Additionally, the woman’s perception of her partner’s dissatisfaction with their sexual relationship is taken into account. The fourth factor, named Anal Sexuality, includes desires, frequency, arousal, and orgasm achieved during anal intercourse. Cronbach’s alpha ranged from excellent (0.95) for Dyadic Sexuality to very good (0.85) for Solitary Sexuality and 0.80 for Anal Sexuality, to acceptable (0.73) for Sexual Difficulties. The test–retest reliability of the instrument was evaluated over a one-month period. The results demonstrated high reliability for all factors, with coefficients ranging from *r* = 0.99 for Dyadic Sexuality, *r* = 0.98 for Solitary Sexuality, *r* = 97 for Sexual Difficulties, and *r* = 0.88 for Anal Sexuality. The final version and factor composition are reported in Supplementary material.

While acknowledging the significant differences between male and female sexual responses (Herbenick et al., 2023; Kok, 2004; Bittoni and Kiesner, 2023) it was deemed appropriate to create a male counterpart to the BISF-W, known as the BISF-M, to provide a comprehensive evaluation of normal male sexual function (Panzeri and Raoli, 2010). The rationale behind this choice was to have an instrument comparable to the BISF-W that would allow for the study of couple sexuality and the comparison of male and female sexuality using the same instrument. The BISF-M questionnaire comprises 22 questions, totaling 63 items. Modeled after the BISF-W, in this version includes specific items related to male sexuality, such as nocturnal pollution. Items related to sexual activity problems were modified by the authors according to the masculine gender. “Ejaculation occurred prematurely” has been add; “Vaginal tightness” has been deleted; “Lack of vaginal lubrication” has been substituted by “Difficulty achieving or maintaining an erection,” “Difficulty reaching orgasm” by “Ejaculation not reached or reached with difficulty,” and “Vaginal infection” by “Urogenital infection.” Similar to the BISF-W, the BISF-M questions that consist of a single item (except for the first two, which examine the presence of any permanent or casual partners) provide multiple-choice responses, while those that consist of multiple items provide responses on a multipoint Likert scale (see Taylor et al., 1994 for a complete view). Cronbach’s alpha for the four factors identified by Panzeri et al. (2009) varied from excellent (0.95 for Dyadic Sexuality) to very good (0.89 for Solitary Sexuality and 0.83 for Anal Sexuality) to appropriate (0.75 for Sexual Difficulties) in the a sample of 190 Italian men. The Supplementary material includes the Italian version of BISF-M with factor composition (S1.3).

The use of the same measuring instrument, in both the female and male versions, aims to fulfill a dual purpose: on the one hand, that of providing important information on male sexual function at different moments in the life cycle (youth, adulthood, old age), thus allowing comparison in qualitative and quantitative terms between the sexuality of women and that of men; on the other hand, to study sexuality within the couple, to evaluate whether and how it changes in relation to the duration of the love relationship, during particular moments, such as status of pregnancy, the menopause, as well as in difficult situations that many couples have to face (sterility, pathologies, etc.).

Sexological and medical literature show contrasting results regarding the overlap between the experience of physical and psycho-emotional sexual arousal in males and females (Chivers and Bailey, 2005). Therefore, an instrument that can compare different genders experiences, emphasizing both the physical and the psycho-emotional experience is essential in therapeutic assessment.

The research cited above provides ample evidence that sexual functioning over the years has been assessed primarily through self-report instruments from a medical perspective, including the International Index of Erectile Function (IIEF; Rosen et al., 1997) and the Female Sexual Function Index (FSFI; Rosen et al., 2000), along with the algorithm developed by Mazer et al. (2000) for scoring the BISF that is based on the linear model of sexual response (American Psychiatric Association (APA), 2000; Kaplan, 1974, 1979; Masters and Johnson, 1966). The aforementioned instruments have concentrated primarily on the physiological aspects of sexuality, while the psychological and relational aspects have been largely overlooked. The BISF is a useful instrument for filling this gap, particularly when considering the four factors analysis by Panzeri and colleagues. This analysis reflects a more psychological perspective, similar to that of other, more recent instruments used for assessing sexual functioning, including the Sexual Desire Inventory (SDI; Spector et al., 1996) and the Orgasm Rating Scale (ORS; Mah and Binik, 2001). Due to the absence of a concise self-evaluation index for male and female sexual function in Italian literature, the objective of this study is to fully validate the BISF-W and BISF-M in the Italian language. In previous studies, the Italian version of the BISF-W was only analyzed using exploratory factor analysis (EFA), whereas the BISF-M was only evaluated using the Cronbach alpha reliability coefficient. Also, in the other validation (Han et al., 2014; Taylor et al., 1994) only EFA was performed. Therefore, we decided to perform different CFA to check which model fits the data better.

There has been an increasing focus on sexuality in the elderly population, as evidenced by recent studies (Štulhofer et al., 2019; Syme et al., 2018). Research has also explored the relationship between age and sexual changes in both men and women (Janssen et al., 2008; Pappalardo and Panzeri, 2015; Pinxten and Lievens, 2014). It was expected that the 4-factor structure (Panzeri et al., 2009) would hold in Italian and that partial factor invariance between men and women would be supported by confirmatory factorial analyses (CFA). We expected to find evidence of partial factor invariance between different age ranges.

We anticipated gender-related differences in the BISF. While males should score higher on the Dyadic, Solitary, and Anal Sexuality BISF factors, females should score higher on the Sexual Dissatisfaction BISF factor. Evolutionary psychology theories propose that innate gender differences in sexual behavior account for this variability (Bjorklund and Kipp, 1996; Ferrucci et al., 2016; Fontanesi and Renaud, 2014; Shackelford and Goetz, 2007; Symons, 1980). Age differences in the BISF were expected due to the negative impact of age on sexuality (Bancroft, 2009; Janssen et al., 2002). According to Bancroft (2009), younger participants are expected to have higher scores in the BISF factors Dyadic, Solitary, and Anal Sexuality, while older participants are expected to have higher scores in the BISF factor Sexual Dissatisfaction due to the decrease in testosterone in men and the adverse effects of menopause in women.

Study 1 presents three confirmatory factor analyses (CFA) of the BISF-W’s scales, involving a sample of healthy women. The study analyzes the factorial structure proposed by Taylor et al. (1994) and Mazer et al. (2000), as well as the Italian 4-factor model (Panzeri et al., 2009). In Study 2, we conducted a confirmatory factor analysis on the same three models proposed for the BISF-W to test an adaptation of the BISF for the male population. The external validity will be examined using two instruments with appropriate psychometric properties to assess sexual function in men (IIEF; Rosen et al., 1997) and women (FSFI; Rosen et al., 2000), while the Quality of Marriage Index (QMI; Norton, 1983) will be used to assess divergent validity. Reliability will be assessed for both the BISF-W and the BISF-M. Study 3 aims to verify the structural invariance of the 4-factor model for both gender and age, as well as to investigate scores by gender.

This study aims to fully validate the Brief Index of Sexual Functioning (BISF), originally developed by Taylor et al. (1994) for a female population (BISF-W), while Panzeri and Raoli developed a male version of the instrument in 2010. So far, research on such a tool has only shown exploratory factor analysis at best (Han et al., 2014; Taylor et al., 1994), and reliability indices that are often not good enough (Han et al., 2014; Mazer et al., 2000; Taylor et al., 1994). We would like to test the different models presented in the literature with a confirmatory analysis, investigating their validity for both men and women. This would allow us to study couples with the same instrument and to compare male and female sexuality.

## 2 Study 1: the BISF-W

### 2.1 Materials and methods

#### 2.1.1 Participants

The sample recruited for the CFA consisted of 6,355 women aged between 18 and 65 years old (*M* = 34.94, SD = 10.52). The inclusion criteria stipulated that applicants were required to be at least 18 years of age, demonstrate proficiency in reading and spoken Italian, and not present any intellectual disabilities. Of the initial questionnaires, 15.44% (*n* = 981) were eliminated due to incoherence (*n* = 713) or containing more than 10% of omissions (*n* = 286). Table 1 presents the demographic and personal characteristics of the final sample of 5,374 Italian women, along with the subsamples used for divergent (*n* = 264) and convergent validity (*n* = 270).

**Table 1:** Demographic characteristics of participants (women).

		CFA sample	Convergent validity sample	Divergent validity sample
		*n* = 5,374	*n* = 265	*n* = 260
		[*n* (%)]	[*n* (%)]	[*n* (%)]
Age	Mean (SD)	34.94 (10.52)	40.42 (15.03)	36.15 (11.91)
	Range	18–65	18–65	18–65
	Missing values	0 (0.0)	0 (0.0)	0 (0.0)
Marital status	Married/Cohabitant	1,617 (30.1)	57 (21.5)	53 (20.4)
	Separated/Divorced	266 (4.9)	95 (35.8)	96 (36.9)
	Widower	42 (0.8)	8 (3.0)	5 (1.9)
	Unmarried	956 (17.8)	85 (32.1)	65 (25.0)
	Missing values	2,493 (46.4)	20 (7.5)	41 (15.8)
Education	Primary School	191 (3.6)	10 (3.8)	25 (9.6)
	Middle School	619 (11.5)	31 (11.7)	15 (5.8)
	Professional School	737 (13.7)	51 (19.2)	50 (19.2)
	High School	1879 (35.0)	69 (26.0)	81 (31.2)
	Degree	1,142 (21.3)	90 (34.0)	78 (30.0)
	Postgraduate degree	124 (2.3)	8 (3.0)	7 (2.7)
	Missing values	682 (12.7)	6 (2.3)	4 (1.5)
Children	Yes	2,383 (44.3)	135 (50.9)	112 (43.1)
	No	2,928 (54.5)	130 (49.1)	115 (44.2)
	Missing values	63 (1.2)	0 (0.0)	33 (12.7)
Sexual experience	Etherosexual	5,225 (97.2)	252 (95.1)	248 (95.4)
	Bisexual	41 (0.8)	2 (0.8)	1 (0.4)
	Homosexual	64 (1.2)	3 (1.1)	3 (1.2)
	Missing values	44 (0.8)	8 (3.0)	8 (3.1)
Sexual activity	Yes	5,115 (95.2)	245 (92.5)	260 (100.0)
	No	250 (4.7)	20 (7.5)	0 (0.0)
	Missing values	9 (0.2)	0 (0.0)	0 (0.0)
Sexual partner	Yes	4,870 (90.6)	235 (88.7)	207 (79.6)
	No	455 (8.5)	28 (10.6)	7 (2.7)
	Missing values	49 (0.9)	2 (0.8)	46 (17.7)

#### 2.1.2 Measures

##### 2.1.2.1 Sociodemographic questionnaire

The following information is provided: age, educational qualifications, marital status, sexual orientation, and the presence of a sexual partner.

##### 2.1.2.2 BISF-W

The Brief Index of Sexual Functioning for Women (BISF-W) Italian translation, as illustrated by Panzeri et al. (2009), consists of 64 items with varying answer options, ranging from Likert scale to multiple choice. Please refer to the accompanying Supplementary material for more details. The Italian adaptation demonstrated an appropriate internal consistency, with Cronbach’s alpha ranging from *α* = 0.95 to *α* = 0.73 (Panzeri et al., 2009). Two algorithms were developed to calculate the number of inconsistent responses to questions related to various aspects of the same activities and the percentage of omissions, as reported in the Supplementary material S1.6. Inconsistent responses were considered omissions. Valid questionnaires were those with less than 10% omissions. It has been decided to allow for a 10% margin of error in the form of omissions or incoherent answers. This is because individuals who do not engage in certain activities, such as oral or anal sex, may choose not to answer those questions. However, this behavior is not consistent throughout the test. Only questionnaires with no missing items on the 46 items considered for the CFA were included.

##### 2.1.2.3 FSFI

The Female Sexual Function Index [FSFI, Rosen et al., 2000; Italian adaptation by Filocamo et al. (2014)] is a multidimensional self-report measure used to assess female sexual functioning. The FSFI consists of 19 items that assess six domains: Sexual Desire, Sexual Arousal, Lubrication, Orgasm, Satisfaction, and Pain. The total score is the sum of all subscale scores. Each item is rated on a scale ranging from 0 to 5 or 1 to 5. The Italian adaptation demonstrates high internal consistency, with Cronbach’s alpha ranging from *α* = 0.92 to *α* = 0.97 for the total sample (Norton, 1983). In this study, internal consistency was appropriate, with Cronbach’s alpha ranging from *α* = 0.74 to *α* = 0.94.

##### 2.1.2.4 QMI

The Quality of Marriage Index (QMI), developed by Norton (1983), is a six-item measure used to assess satisfaction levels. Higher scores on the QMI indicate higher levels of satisfaction. The items in this measure evaluate overall satisfaction and are rated on 6- or 10-point Likert scales. The QMI demonstrates high internal consistency, as evidenced by Cronbach’s alpha of 0.96. Although there is no Italian validation of this instrument, it is often used in the literature (e.g., Bonechi and Tani, 2011). The present study found high internal consistency, with a Cronbach’s alpha of 0.94.

#### 2.1.3 Procedure

The data collection occurred from 24 April 2018 to 28 May 2022. Participants were recruited in person by psychology master’s students from universities, wellness centers, sports facilities, and recreational centers. Participants were informed of the research’s objectives and privacy policies. They did not receive any financial compensation for their participation in the study. Each participant gave their written consent for the study by responding to a specific item. The protocol was completely anonymous. All participants answered the sociodemographic questionnaire and the BISF in a paper and pencil format, which has been proved to be a reliable source of collecting information (Dillman et al., 2014). Different subsamples completed questionnaires to measure convergent or divergent validity. The time taken to complete the various questionnaires ranged from 15 to 30 min. The research protocol was approved by the Ethical Committee of Psychological Research of *blinded* University protocol 2,615.

#### 2.1.4 Statistical analysis

LISREL was used to conduct overall confirmatory factor analysis (CFA) analyses, on all items except for questions 1, 2, 21, and 22, which are independent variables (as in Taylor et al., 1994). Several items showed slightly skewed distributions, with skew values ranging from −1.23 to 2.93 (median = 0.23). Furthermore, the kurtosis values ranged from −1.64 to 8.65 with a median of −0.55. Based on this evidence, we chose to use a maximum likelihood estimator that is robust and starts from the asymptotic variance and covariance matrix (Jöreskog and Sörbom, 1996). Three CFA models were tested to achieve the main study goals. The CFA that was primarily tested followed the structure obtained from an EFA in a previous study by Panzeri et al. (2009). A correlated factors model with four factors was tested using 47 raw variables was tested with correlated errors between the same item in different questions. In particular, the items “Erotic Kissing,” “Masturbation alone,” “Mutual masturbation,” “Petting and foreplay,” “Oral sex (giving or receiving)” and “Vaginal penetration or intercourse” reported in questions 4, 5, 7 and 11; the item “Sexual fantasy” reported in questions 5, and the item “Sexual anxiety” reported in questions 6 and 13. Additionally, we tested two alternative CFA models based on the literature: (a) a model with three factors from 15 subtotals as suggested by Taylor et al. (1994); and (b) a model with seven factors from 17 subtotals as proposed by Mazer et al. (2000). The overall fit of these models was evaluated based on standard fit index criteria (Hu and Bentler, 1999). This included Satorra and Bentler scaled Chi-square (χ2, with a desired non-significance), Root Mean Square Error of Approximation (RMSEA, with desired values of ≤0.06), Standardized Root Mean Square Residual (SRMR, with desired values of ≤0.08), and Comparative Fit Index and Non-Normed Fit Index (CFI and NNFI, with desired values of ≥0.95). We evaluated the model’s fit to the data using RMSEA and SRMR values ranging from 0.05 to 0.08, and CFI and NNFI values ranging from 0.90 to 0.95. These values indicate a satisfactory fit. In calculating the sample size, we adhered to the established best practices for factor analyses and collected a sufficient number of samples to achieve a 20:1 ratio of participants to scale items (e.g., Carpenter, 2018; Kline, 2013). This sample size ensured reliable results for our newly developed Hero’s Journey Scale, which further helped minimize measurement error, thereby reducing the potential for Type II errors (Asendorf et al., 2013).

The Statistical Package for the Social Sciences (SPSS) version 26 for Windows was used for all other statistics. Pearson’s r was used to calculate correlations between the BISF subscales, the FSFI dimensions, and the QMI. McDonald’s omega was used to assess internal consistency for all subscales and the total score. An alpha value greater than 0.90 is considered excellent, while values between 0.80 and 0.90 are very good. Values between 0.70 and 0.80 are appropriate, values between 0.60 and 0.70 are sufficient, and values less than 0.60 are insufficient indicators.

### 2.2 Results

#### 2.2.1 Confirmatory factor analysis

Table 2 reports fit indices indicating that the four-factor model proposed by Panzeri et al. (2009) is the best overall model for women. Although the seven-factor model shows an adequate fit, it is lower than the four-factor model. On the other hand, the three-factor model shows poor fit. Factor loadings of the 4-factor model are reported in Supplementary Table S2.1.

**Table 2:** Fit Index of 4 factor model (Panzeri et al., 2009), 3 factor model (Taylor et al., 1994) and 7 factor model (Mazer et al., 2000).

Model	χ2	df	χ2/df	RMSEA	GFI	NFI	NNFI	CFI	SRMR
4 factor	28869.34	979	29.49	0.073	0.80	0.95	0.95	0.95	0.068
3 factor	7477.87	81	92.32	0.130	0.83	0.90	0.87	0.90	0.110
7 factor	4881.43	99	49.31	0.095	0.90	0.93	0.93	0.95	0.076

χ^2^, chi square; df, degree of freedom; RMSEA, root mean square error of approximation; BIC, Bayesian information criterion; GFI, goodness of fit index; NFI, normed fit index; NNFI, non normed fit index; CFI, comparative fit index.

#### 2.2.2 Reliability

The internal consistency analysis revealed an overall McDonald’s *ω* coefficient of 0.943 for BISF-W. The Cronbach *α* coefficient for the four subscales of BISF-W were as follows: 0.957 for Dyadic Sexuality, 0.866 for Solitary Sexuality, 0.731 for Sexual Difficulties, and 0.917 for Anal Sexuality.

#### 2.2.3 Correlation among BISF-W scales and with age

A positive correlation was found between the four BISF-W factors. Each factor is correlated with other subscales. Dyadic Sexuality moderately positively correlated with Solitary Sexuality (*r* = 0.41, *p* < 0.001) and weakly correlated with Anal Sexuality (*r* = 0.31, *p* < 0.001). Solitary Sexuality weakly positively correlated with Anal Sexuality (*r* = 0.32, *p* < 0.001). There was a weak negative correlation between age and Dyadic and Solitary Sexuality, with respective correlation coefficients of *r* = −0.25 (*p* < 0.001) and *r* = −0.23 (*p* < 0.001).

#### 2.2.4 Convergent and divergent validity

Table 3 displays the Pearson’s correlation among the BISF-W factors, FSFI dimensions, and the QMI. The Pearson’s correlation among the BISF-W factors, FSFI dimensions, and the QMI is presented in Table 3. The results demonstrated that dyadic sexuality exhibited robust correlations with the Orgasm and Pain dimensions of the FSFI, while moderate correlations were observed with the Sexual Arousal and Lubrication dimensions. In contrast, solitary sexuality demonstrated a moderate correlation with the Orgasm dimension and weaker correlations with the Sexual Arousal and Pain dimensions. Sexual difficulties exhibited moderate correlations with all FSFI dimensions, with the exception of Sexual Desire, and a negative correlation with the QMI. Conversely, Anal Sexuality demonstrated no correlation with any FSFI dimensions or with the QMI.

**Table 3:** Correlations between BISF-W, FSFI, and QMI factors.

		FSFI (*n* = 265)						QMI (*n* = 260)
		Desire	Arousal	Lubrication	Orgasm	Satisfaction	Pain	Total
BISF-W	Dyadic	–0.06	0.36*	0.31*	0.62*	–0.07	0.67*	–0.01
	Solitaire	–0.06	0.20*	0.14	0.37*	0.08	0.29*	−0.16
	Difficulties	0.07	0.39*	0.34*	0.31*	0.29*	0.31*	−0.24*
	Anal	−0.13	-,02	,07	,09	−0.14	0.16	–0.12
								

*p < 0.001, BISF-W, Brief Index of Sexual Functioning for Women (Panzeri et al., 2009); FSFI, Female Sexual Function Index (Rosen et al., 2000); QMI, Quality of Marriage Index (Norton, 1983).

### 2.3 Discussion

A confirmatory factor analysis (CFA) confirmed that a four-factor model (Dyadic Sexuality, Solitary Sexuality, Sexual Difficulties, and Anal Sexuality) fit the data best, outperforming three- and seven-factor models. Reliability was high across all the subscales. Age negatively correlated with Dyadic and Solitary Sexuality, indicating a decline in sexual functioning with age. Correlations were observed between BISF-W factors, with Dyadic Sexuality correlating moderately with Solitary Sexuality and Anal Sexuality. Anal Sexuality showed no significant correlations with FSFI or QMI. The moderate correlation between dyadic and solitary sexuality may be explained by the fact that, according to the literature, women that are in a romantic relationship may engage in more compensatory autoerotic behavior that those who are not (Dekker and Schmidt, 2013; Huang et al., 2022; Pinkerton et al., 2003) to make up for an unsatisfactory sex life with their partner. This might explain the positive correlation found. Finally, regarding Anal Sexuality, it appears to be a distinct category that is correlated with Dyadic Sexuality. Across different ages, anal sex seems to raise a lot of concerns regarding coercion and health risks (Pickles et al., 2023), cultural expectations and social norms (Fahs and Swank, 2021) and it perceived to be related to men’s sexual entitlement (Fahs and Swank, 2021). For these reasons, anal sexuality can be a controversial and undiscussed topic for women, which seems to be performed mostly in meaningful and trustful relationship, in fact, as suggested by Reynolds et al. (2015), some women considered anal sex as more intimate than vaginal sex, and only engage in it with specific partners. In light of the aforementioned points, it would be beneficial to investigate the influence of sexual education derived from pornography culture and its correlation with anal sexuality. Furthermore, it would be beneficial to investigate the potential for homophobia related to this practice in men.

## 3 Study 2: the BISF-M

### 3.1 Materials and methods

#### 3.1.1 Participants

A total of 2,585 Italian men were recruited for the study; 378 questionnaires were excluded from the analysis due to incoherence or omissions, leaving a total of 2,207 valid responses. In order to be eligible for inclusion in the study, participants were required to meet the following criteria: they had to be at least 18 years of age, possess the ability to read and speak Italian, and not have any intellectual disabilities. Table 4 presents the demographic and personal characteristics of the total sample of 2,207 Italian men, as well as the subsamples used for divergent (n = 259) and convergent validity (*n* = 453).

**Table 4:** Demographic characteristics of participants (men).

		CFA sample	Convergent validity sample	Divergent validity sample
		*n* = 2,207	*n* = 445	*n* = 257
		[*n* (%)]	[*n* (%)]	[*n* (%)]
Age	Mean (SD)	38.25 (13.67)	41.16 (16.92)	31.43 (9.08)
	Range	18–80	18–80	18–62
	Missing values	27 (1.2)	1 (0.2)	0 (0.0)
Marital status	Married/Cohabitant	1,125 (51.0)	195 (43.8)	76 (29.6)
	Separated/Divorced	122 (5.5)	31 (7.0)	13 (5.1)
	Widower	18 (0.8)	4 (0.9)	0 (0.0)
	Unmarried	922 (41.8)	214 (48.1)	167 (65.0)
	Missing values	20 (0.9)	1 (0.2)	1 (0.4)
Education	Primary School	44 (2.0)	16 (3.6)	0 (0.0)
	Middle School	288 (13.0)	34 (7.6)	9 (3.5)
	Professional School	320 (14.5)	80 (18.0)	54 (21.0)
	High School	975 (44.2)	163 (36.6)	101 (39.3)
	Degree	494 (22.4)	142 (31.9)	88 (34.2)
	Postgraduate degree	75 (3.4)	10 (2.2)	5 (1.9)
	Missing values	11 (0.5)	0 (0.0)	0 (0.0)
Children	Yes	934 (42.3)	192 (43.1)	69 (26.8)
	No	1,292 (57.2)	249 (56.0)	185 (72.0)
	Missing values	11 (0.5)	4 (0.9)	3 (1.2)
Sexual experience	Etherosexual	2,107 (95.5)	417 (93.7)	242 (94.2)
	Bisexual	41 (1.9)	18 (4.0)	8 (3.1)
	Gay/Lesbian	39 (1.8)	8 (1.8)	6 (2.3)
	Missing values	20 (0.9)	2 (0.4)	1 (0.4)
Sexual activity	Yes	2,169 (98.3)	431 (96.9)	256 (99.6)
	No	33 (1.5)	14 (3.1)	1 (0.4)
	Missing values	5 (0.2)	0 (0.0)	0 (0.0)
Sexual partner	Yes	1985 (89.9)	390 (87.6)	233 (90.7)
	No	221 (10.0)	55 (12.4)	24 (9.3)
	Missing values	1 (0.0)	0 (0.0)	0 (0.0)

#### 3.1.2 Measures

##### 3.1.2.1 Sociodemographic questionnaire

The following information is collected: age, marital status, educational attainment, sexual orientation, and the presence of a sexual partner.

##### 3.1.2.2 BISF-M

The Brief Index of Sexual Functioning for Men (BISF-M), illustrated above (Panzeri and Raoli, 2010), is composed of 66 items with different response options, varying from Likert-scale to multiple-choice. For more information, please refer to the supporting materials. The internal consistency of the data is appropriate, as evidenced by Cronbach’s ranging from *α* = 0.95 to *α* = 0.75.

The same algorithms utilized in Study 1 were employed to quantify the number of inconsistent responses and omissions, as reported in the Supplementary material S1.7 and only questionnaires with no missing items on the 46 items considered for the CFA were included.

##### 3.1.2.3 IIEF

The International Index of Erectile Function (IIEF), developed by Rosen and colleagues in 1997, is a self-report questionnaire consisting of 15 items. It is used to evaluate erectile dysfunction and related factors. It examines five interconnected domains: Erectile Function, Orgasmic Function, Sexual Desire, Intercourse Satisfaction, and Overall Satisfaction. The total score is calculated by summing the subscale scores. Each item is scored from 0 to 5. The test demonstrates strong internal consistency, with Cronbach’s values of 0.73 or higher for the five main domains and 0.91 or higher for the total scale. The study used an Italian adaptation of the IIEF that was widely used throughout the country (e.g., Rosen et al., 1997). In this study, the internal consistency was appropriate, with Cronbach’s alpha ranging from *α* = 0.85 to *α* = 0.95.

##### 3.1.2.4 QMI

The QMI (Norton, 1983) described above, had a high level of internal consistency for men, with Cronbach’s alpha = 0.96, as was found in the current study.

#### 3.1.3 Procedure

The data collection for this study occurred between 3 March 2019 and 25 May 2022 using a paper and pencil format. The procedure was identical to that of study 1. Pearson’s *r* was used to calculate correlations between the BISF subscales, the IIEF dimensions, and the QMI.

#### 3.1.4 Statistical analysis

The statistical analyses in this study were identical to those in Study 1.

### 3.2 Results

#### 3.2.1 Confirmatory factor analysis

Table 5 reports fit indices indicating that the four-factor model proposed by Panzeri et al. (2009) is the best overall model for men. As in women the seven-factor model show a lower fit than the four-factor model even if both models show lower fit indices in men than in women. However, the three-factor model shows a poor fit in both men and women. Factor loadings of the 4 factor model are reported in table S2.2.

**Table 5:** Fit Index of 4 factor model, 3 factor model (Taylor et al., 1994) and 7 factor model (Mazer et al., 2000).

Model	χ2	df	χ2/df	RMSEA	GFI	NFI	NNFI	CFI		SRMR
4 factor	15262.89	981	15.56	0.081	0.75	0.93	0.92	0.93		0.092
3 factor	1633.98	81	20.17	0.093	0.89	0.94	0.92	0.94		0.080
7 factor	2275.36	99	22.98	0.100	0.87	0.92	0.89	0.92		0.093

χ^2^, chi square; df, degree of freedom; RMSEA, root mean square error of approximation; BIC, Bayesian information criterion; GFI, goodness of fit index; NFI, normed fit index; NNFI, non normed fit index; CFI, comparative fit index.

#### 3.2.2 Reliability

The BISF-M exhibited excellent internal consistency, as indicated by an overall McDonald’s *ω* coefficient of 0.940. The Cronbach α coefficient for the four subscales of BISF-M was excellent for both Dyadic Sexuality (0.947) and Anal sexuality (0.911), very good for Solitary Sexuality (0.892) and only sufficient for Sexual Difficulties (0.615).

#### 3.2.3 Correlation among BISF-M scales and with age

A positive correlation was observed between the four BISF factors. Each factor correlates positively with the other factors, except for Sexual Difficulties: Dyadic Sexuality correlates positively with Solitary Sexuality (*r* = 0.37, *p* < 0.001) and Anal Sexuality (*r* = 0.41, *p* < 0.001), and weakly with Sexual Difficulties (*r* = 0.17, *p* < 0.001); Solitary Sexuality correlates positively with Anal Sexuality (*r* = 0.36, *p* < 0.001). There is a weak negative correlation between age and Dyadic and Solitary Sexuality (*r* = −0.29, *p* > 0.001 and *r* = −0.38, *p* < 0.001 respectively). However, there is no consistent correlation between age and Sexual Difficulties or Anal Sexuality (*r* = 0.11, *p* < 0.001 and *r* = −0.17, *p* < 0.001 respectively).

#### 3.2.4 Convergent and divergent validity

Table 6 presents the Pearson’s correlation between the BISF-M factors, the IIEF dimensions, and the QMI. Although the BISF-M Sexual Difficulties factor did not correlate with any IIEF dimensions, the BISF-M Dyadic Sexuality factor did correlate with all IIEF dimensions. Similarly, the BISF-M Anal Sexuality factor correlated with all IIEF dimensions except for the Orgasmic Function dimension, and the BISF-M Solitary Sexuality factor only correlated with the IIEF Sexual Desire dimension. The only factor that correlated with the QMI was the BISF-M Dyadic Sexuality factor.

**Table 6:** Correlations between BISF-M, IIEF, and QMI factors.

		IIEF (*N* = 445)					QMI (*N* = 257)
		Erectile function	Orgasmic function	Sexual desire	Intercourse satisfaction	Overall satisfaction	Total
BISF-M	Dyadic	0.70*	0.60*	0.61*	0.76*	0.63*	0.39*
	Solitaire	0.25*	0.25*	0.42*	0.20*	0.09	–0.01
	Difficulties	0.09	0.11	0.02	0.14	–0.07	–0.08
	Anal	−0.31*	0.20*	0.27*	0.30*	0.26*	–0.04

*p < 0.001. BISF-W, Brief Index of Sexual Functioning for Women (Panzeri et al., 2009); IIEF, International Index of Erectile Function (Rosen et al., 1997); QMI, Quality of Marriage Index (Norton, 1983).

### 3.3 Discussion

The study validated the Brief Index of Sexual Functioning for Men (BISF-M) in an Italian sample of 2,207 men, focusing on its reliability and factor structure. The BISF-M, composed of 66 items, was used alongside the International Index of Erectile Function (IIEF) and the Quality of Marriage Index (QMI). The confirmatory factor analysis revealed that the four-factor model (Dyadic, Solitary, Anal Sexuality, and Sexual Difficulties) provided the best fit. Reliability was high for Dyadic, Solitary and Anal Sexuality, though the Sexual Difficulties subscale was only moderately reliable. Age negatively correlated with Dyadic and Solitary Sexuality, indicating a decline in sexual activity over time. The BISF-M demonstrated good convergent validity with the IIEF, except for the Sexual Difficulties subscale, which did not correlate with IIEF dimensions in our sample.

Our findings demonstrate a statistically significant positive correlation between dyadic and solitary sexuality in men. This aligns with existing literature suggesting that sexual relationships can lead to an increase in overall sexual activity, including solitary activities such as masturbation. This pattern may be indicative of a broader phenomenon wherein individuals, regardless of gender, who are sexually active with a partner may also engage in solitary sexual behaviors with greater frequency. For men, this does not necessarily indicate compensatory behavior, as has been suggested for women (Dekker and Schmidt, 2013), but rather an overall heightened sexual desire or arousal, which may result in more frequent sexual activity in general. Some studies have indicated that men who are more sexually active in a dyadic context may also experience an increase in libido, which can extend to solitary behaviors (Pinkerton et al., 2003). The positive correlation between dyadic and solitary sexuality in the male sample indicates that men, like women, may experience reinforcement of their sexual drive through their sexual relationships, resulting in increased solitary sexual activities. In contrast, research has indicated that solitary sexuality in women may occasionally serve a compensatory function when dyadic sexual satisfaction is lower (Dekker and Schmidt, 2013; Huang et al., 2022). This is consistent with findings that male sexual desire is often maintained or enhanced through multiple forms of sexual expression, whether partnered or solitary (Herbenick et al., 2023).

## 4 Study 3: the BISF

### 4.1 Materials and methods

#### 4.1.1 Participants

We have created a comprehensive database that includes data from both males and females. The study population was divided into three age groups: 18–29 (*n* = 1925 women, *n* = 717 men), 30–49 (*n* = 2,736 women, *n* = 970 men), and over 50 (*n* = 686 women, *n* = 493 men). The age groups were determined based on a review of the literature, which indicates that the average age of menopause is 50 years old, while the age at which women typically experience heightened sexual vigor is 30 years old (Harlow et al., 2023; Harris and Vitzthum, 2013; Masters and Johnson, 1966).

#### 4.1.2 Procedure

To ensure accuracy, we have left blank responses to questions that are not applicable to certain genders. For example, questions such as “Vaginal tightness” are only applicable to women, while “Ejaculation occurred prematurely” is only applicable to men. We consider as the same answer questions that, although slightly different, refer to the same sexual response phase, that is “Lack of vaginal lubrication” and “Difficulty achieving or maintaining an erection”; “Difficulty reaching orgasm” and “Ejaculation not reached or reached with difficulty”; “Vaginal infection” and “Urogenital infection.” “Ejaculation occurred prematurely” has been add, “Vaginal tightness” has been deleted.” For the CFA analysis we matched the item 14_0 “Ejaculation occurred prematurely” (only for men) with the items 14_5 “Vaginal tightness” (only for women).

#### 4.1.3 Statistical analyses

LISREL was used to conduct multigroup CFA measurement invariance procedures.

After evaluating the four factors model as the best model, we test the measurement invariance of this model across the six groups generated by crossing age groups and gender. We followed the standard steps for testing measurement invariance, as outlined by Pendergast et al. (2017). This included testing a configural model (an overall model without constraints fit across groups), a metric model (constraining factor loadings to equality across groups), and finally a scalar model (constraining item intercepts and factor loadings to equality across groups). Measurement invariance constraints were evaluated by primarily considering the change in CFI values with a desired decrement in model fit of 0.01 or less, following the method proposed by Cheung and Rensvold (2002). We considered changes in Satorra and Bentler scaled Chi-square and RMSEA when evaluating more restrictive measurement invariance models. Specifically, we aimed for a non-significant Chi-square and a decrement in model fit of 0.01 or less for RMSEA (Chen, 2007).

SPSS version 26 for Windows was used for all other statistics. A Multivariate Analysis of Variance (MANOVA) was conducted to assess differences in BISF factors between age groups for both women and men separately, as well as to evaluate differences in gender and age groups in the total sample. The Bonferroni post-hoc analysis method was utilized to compare age groups.

### 4.2 Results

#### 4.2.1 Invariance

We considered six groups: three age groups for women (*n* = 1925 for the first age group; *n* = 2,763 for the second age group; *n* = 686 for the last age group) and three age groups for men (*n* = 717 for the first age group; *n* = 970 for the second age group; *n* = 493 for the last age group). Table 7 shows that the configural model fits well, and imposing metric invariance constraints only slightly decreased the model fit (ΔCFI = 0.01 and ΔRMSEA = 0.002). Additionally, when scalar invariance constraints were applied, there were relevant reductions in model fit (ΔCFI = 0.04 and ΔRMSEA = 0.013) indicating the presence of significant differences on factor scores among groups.

**Table 7:** Age and Gender measurement invariance fit statistics.

Model	χ2	df	*p*	RMSEA	SRMR	CFI	NNFI	Delta χ2	Delta df	*p*	Delta RMSEA	Delta CFI
Configural (4 factors model)	59,431,97	5,904	<0.001	0,085	0,10	0,93	0,92					
Metric (4 factors model)	64,565,94	6,139	<0.001	0,087	0,12	0,92	0,92	5,133,97	235	<0.001	0,002	0,01
Scalar (4 factors model)	86,581,81	6,374	<0.001	0,100	0,11	0,89	0,89	22,015,87	235	<0.001	0,013	0,03

χ^2^, Satorra and Bentler scaled Chi-square; df, degree of freedom; RMSEA, Root Mean Square Error of Approximation; SRMR, Standardized Root Mean Square Residual; CFI, Comparative Fit Index; NNFI, Non Normed Fit Index; Δχ2 statistics are in reference to the preceding model in the table.

#### 4.2.2 Manova

The MANOVA results for women indicated a significant effect of age groups on all factors [*F*(2,5,371) = 194.04, *p* < 0.0001, partial η^2^ = 0.07 for Dyadic Sexuality, *F*(2,5,371) = 154.21, *p* < 0.001, partial η^2^ = 0.05 for Solitary Sexuality and *F*(2,5,371) = 13.72, *p* < 0.001, partial η^2^ = 0.01 for Anal Sexuality], except for Sexual Difficulties [*F*(2,5,371) = 2.51, *p* = 0.081, partial η^2^ < 0.01]. *Post-hoc* analysis indicated a progressive and always significant decrease, from the first (18–29 years) to the last age group (50+ years). For men the MANOVA results showed a significant effect of age groups for all factors [*F*(2,2,177) = 103.16, p < 0.001, partial η^2^ = 0.09 for Dyadic Sexuality, *F*(2,2,177) = 154.51, *p* < 0.001, partial η^2^ = 0.12 for Solitary Sexuality, *F*(2,2,177) = 43.36, *p* < 0.001, partial η^2^ = 0.04 for Anal Sexuality and *F*(2,2,177) = 11.09, *p* < 0.001, partial η^2^ = 0.01 for Sexual Difficulties]. *Post-hoc* analysis showed no significant difference between the first age group (18–29 years) and the second age group (30–49 years) for all factors except the Solitary sexuality one. There was a significant decrease in all factors except Sexual Difficulties, which increased, from the second age group to the last age group (50+ years). Solitary sexuality decreased significantly with age (see Supplementary Table S1).

A MANOVA was conducted on both male and female participants, and the results showed a significant age effect for all factors [*F*(2,7,548) = 249.17, *p* < 0.001, partial η^2^ = 0.06 for Dyadic Sexuality, *F*(2,7,548) = 315.85, *p* < 0.001, partial η^2^ = 0.08 for Solitary Sexuality, *F*(2,7,548) = 78.25, *p* < 0.001, partial η^2^ = 0.02 for Anal Sexuality and *F*(2,7,548) = 2.54, *p* = 0.003, partial η^2^ < 0.01 for Sexual Difficulties]. *Post-hoc* analysis showed a significant and progressive decrease in all factors, except for Sexual Difficulties, from the first age group (18–29 years) to the last age group (50+ years). There was only a significant increase in sexual difficulties from the first age group (18–29 years) to the second age group (30–49 years). Additionally, a significant interaction between gender and age groups was found for all factors [*F*(2,7,548) = 8.66, *p* < 0.001, partial η^2^ < 0.01 for Dyadic Sexuality, *F*(2,7,548) = 12.75, *p* < 0.001, partial η^2^ < 0.01 for Solitary Sexuality, *F*(2,7,548) = 35.34, *p* < 0.001, partial η^2^ = 0.01 for Anal Sexuality and *F*(2,7,548) = 2.53, *p* = 0.003, partial η^2^ < 0.01 for Sexual Difficulties]. *Post-hoc* analysis showed no significant gender effect for Sexual Difficulties in the last age group (50+ years). There was no significant difference in Dyadic and Anal activity between the first (18–29 years) and the second age group (30–49 years) for men, as found in the MANOVA for men. Similarly, there was no significant difference in Sexual Difficulties between age groups for women, as found in the MANOVA for women.

### 4.3 Discussion

The results of the invariance analysis showed that the configural model fit well across six age and gender groups, with only a slight reduction in fit when metric invariance constraints were applied. However, applying scalar invariance resulted in significant reductions in fit, indicating differences in factor scores across age groups. The MANOVA results for women revealed a significant age effect on all factors, with sexual functioning decreasing progressively with age, except for Sexual Difficulties, which remained constant. For men, similar age effects were observed, with sexual functioning decreasing with age, except for Sexual Difficulties, which increased from the second to the last age group. A combined MANOVA for both genders confirmed these trends and also highlighted an interaction between gender and age for all factors. No significant gender effect was found for Sexual Difficulties in the oldest age group, and both Dyadic and Anal Sexuality showed no significant differences between younger and middle-aged men.

## 5 General discussion

The study presents a fully psychometric validation of the Italian version of the BISF questionnaire. This measure was initially developed by Taylor et al. (1994) to assess women’s sexuality. This study proposes a BISF version that enables for an evaluation of sexual functioning in both men and women.

The CFA indicated that the fit indexes for the four-factor model are superior to those of the three-factor or seven-dimension models for both BISF-W and BISF-M. The correlation between factors was in the desired direction, and the internal consistency was good, according to the literature (Taber, 2018). This makes them reliable tools for assessing sexual functioning in both women and men. Previous American studies have identified factors or dimensions related to a psychophysiological model of the sexual response cycle (Mazer et al., 2000; Taylor et al., 1994). The present study revealed four factors - Dyadic Sexuality, Solitary Sexuality, Sexual Difficulties, and Anal Sexuality—which appear to reflect a more psychological and comprehensive model that distinguishes solo auto-erotic activities from those performed as a couple. Indeed, other validated tools, such as the Sexual Desire Inventory (SDI, Spector et al., 1996) or the Orgasm Rating Scale (ORS, Mah and Binik, 2002), distinguish between dyadic sexual desire and solitary sexual desire, or the subjective experience of an orgasm in the context of sexual relationships and solitary masturbation, respectively. Dyadic Sexuality is emerging as the most important factor, explaining a higher percentage of the variance than the others. This finding is consistent with the literature, which suggests that relational intimacy plays a mediating role in sexual functioning (Basson, 2000; Janssen et al., 2008; McCabe et al., 2010; Witherow et al., 2017).

The factor structure of the BISF did not show full scalar invariance across age groups for both genders and the global sample (men + women), reaching only metric invariance according to our analyses. This result indicated that there were differences in both gender and age groups. These differences could be further analyzed using MANOVA. The MANOVA results confirm that there are different scores on the factors in each age group. Specifically, sexual functioning decreases with age while sexual problems increase only in men. This is in line with the literature that recognizes age as the primary risk factor for sexual function (Han et al., 2014; Hayes and Dennerstein, 2005). For example, a decrease in testosterone (which affects body image and increases the risk of sexual dysfunction) or cognitive decline has been seen to be related to sexual problems (Barone et al., 2022; Tavares et al., 2020). Moreover, the literature on the variation of female sexual functioning with age is contrasting. While it is certain that age influences the physiological aspects of sexuality, such as lubrication or pelvic floor health, with regard to psycho-emotional aspects there is a great deal of intra- and inter-individual variability that makes it difficult to generalize the findings concerning the relationship between age and female sexual function (Athey et al., 2021). Nevertheless, the BISF is able to measure the same constructs (Dyadic Sexuality, Solitary Sexuality, Sexual Difficulties, and Anal Sexuality) in the same way across different groups by achieving configural and metric invariance. This ensures that the relationships between items and their underlying factors are consistent, thereby allowing for valid comparisons of factor structures and associations between factors across groups. The absence of scalar invariance suggests that although the constructs are assessed in a comparable manner, the absolute levels of these constructs may vary due to group-specific biases or discrepancies in response styles. Nevertheless, the findings of our study on gender and age differences in sexual functioning offer valuable insights into the ways in which these constructs manifest differently across groups. The comparisons made in the study have important practical implications for clinical and therapeutic settings. For instance, understanding that men score higher on Dyadic, Solitary, and Anal Sexuality while women score higher on Sexual Difficulties can inform tailored interventions and therapeutic approaches that address the specific needs and challenges faced by different groups.

Our analyses did not support the scalar invariance of the factor structure of the BISF across genders. The MANOVA results confirm that there are different factor scores for women and men. As expected, men scored higher in Dyadic, Solitary, and Anal sexuality, while women scored higher in Sexual difficulties. These results may be related to gender roles and the differences in the perception of sex and sexuality across genders. It is possible that since women continue to have some taboos related to expressing themselves about sex and sexuality (Farvid et al., 2017), particularly masturbation, respond to question about their sexual behavior with neutrality, without over-exposing themselves with respect to topics such as masturbation. In contrast, it may be easier for them to talk in terms of problems and health, since they are more aware of their bodies and any alterations in sexual experience. On the other hand, men are less willing to recognize, talk, and seek help for psychological issues, including sexual problems (Liddon et al., 2018), and that may be the explanation for the gender differences in the scores for sexual difficulties. Further qualitative studies are needed to deem these matters.

The reliability of the 4-factor model for both BISF-W and BISF-M appears to be excellent or adequate, surpassing that of the previous model (Mazer et al., 2000; Taylor et al., 1994), which was insufficient for many factors and dimensions. The convergent validity correlations appeared strong enough for most variables for both the BISF-W and the BISF-M. The correlation between the BISF-W Sexual Difficulties factor and FSFI Satisfaction is noteworthy. One possible explanation could be that for women, sexual satisfaction is more closely linked to dyadic factors such as intimacy and caring, which can help them face sexual physical problems such as poor lubrication or pain during intercourse, or even anxiety (Leavitt et al., 2021; Panzeri, 2023). Women can experience sexual satisfaction even under these conditions, whereas men seem to require sexual performance to feel sexually satisfied (Basson, 2001; Leavitt et al., 2021; Panzeri, 2023). Further studies are necessary to settle this issue. In terms of divergent validity, there was only a correlation found between the Dyadic factor of the BISF-M and the QMI, as opposed to any other factors between the two. This correlation may be explained by the fact that couple sexual activity is very important for men in order to perceive a good quality of the relationship, while daily attentions and emotional intimacy play the same role for women (Johnson and Zuccarini, 2010). Nevertheless, both instruments share the same structure (Dyadic, Solitary, Sexual Difficulties, and Anal Sexuality), which allows for direct gender comparisons in clinical and non-clinical settings. The potential for comparing male and female sexuality using a single framework makes the BISF-W and BISF-M highly valuable for studying couple dynamics.

These initial findings are highly relevant, as they provide insight into how both men and women experience their sexuality beyond the physiological aspects considered by the sexual response cycle. Overall, the Brief Index for Sexual Functioning is a reliable measure that provides information on the sexual behavior and satisfaction of both men and women throughout different phases of life and stages of relationships. At the same time, it enables the comparison of male and female sexual functioning in specific moments of a couple’s life, such as pregnancy, postpartum, menopause, widowhood, infertility, or other medical conditions. Moreover, for what concern the external validity of the instrument, the study included a large and diverse sample of 6,355 women and 2,207 men, aged 18–80, from various educational backgrounds and marital statuses. This broad demographic range enhances the generalizability of the results to the Italian population of the BISF. Also, the study found significant differences in sexual functioning across different age groups and between genders. These findings align with existing literature, suggesting that the BISF can reliably capture variations in sexual functioning across different demographic groups. The BISF appears to be a suitable measure to assess sexuality both in clinical and non-clinical populations and can be a valuable tool in the field of human sexuality, when used in conjunction with other tools. It can provide valid and reliable information for operators working in this field to establish appropriate prevention and intervention programs, both in longitudinal and cross-sectional research. In particular, for what pertain sexual therapy and couple therapy, the BISF can help patients to express their concerns or difficulties in the evaluation stage, thereby overcoming any embarrassments or taboos that one is not yet ready to talk about directly. Moreover, due to its reliability characteristic, it can use as a valuable measure to evaluate the course of treatment *in itinere*, highlighting any changes in patients. It could also be applicable to non-binary and transgender populations, referring to the version that pertains to current anatomy, but would require further validation in this specific population.

### 5.1 Limitations and future directions

This study has some limitations. This study was done only on the general population. It may be useful to administer the BISF to a sample of men and women with sexual dysfunction. Moreover, due to the small number of non-heterosexual participants, it was not possible to conduct adequate psychometric analyses regarding the validity of the instrument in sexually diverse samples. Future studies should verify our results in this type of population as well. The administration of paper and pencil tests suffers from many omissions. In future studies, however, we can avoid such problems by using an online administration where all answers are mandatory, and participants can stop the compilation at any time without negative consequences. The present study compared gender and age only form a quantitative point of view. Future research on sexual functioning should employ qualitative research methods, such as focus groups, to assess sexual functioning across various age groups. Finally, while the BISF provides valuable insights into sexual functioning, the lack of scalar invariance necessitates careful consideration when applying and interpreting the instrument measurements in clinical setting. By contextualizing findings, using complementary measures, incorporating qualitative insights, and being aware of patients’ response styles, clinicians can make more informed decisions based on BISF data.

## 6 Conclusion

The Brief Index of Sexual Functioning (BISF) is a questionnaire that can efficiently assess sexual functioning in the general population. Its good psychometric qualities make it a suitable tool for study and screening in Italy. The validation of the BISF-W in Italian and the adaptation of the BISF-M for a male sample demonstrated strong psychometric properties for assessing sexual functioning in both genders. To date, the Brief Sexual Functioning Inventory (BISF) is one of the few published and useful tools for assessing sexual functioning in both genders from a psychological perspective. It can be easily used in research protocols to assess sexual behavior, desire, and fantasies, as well as in a clinical setting. This validated tool will be useful for evaluating sexual functioning, providing valid and reliable information to operators in this field to set up appropriate prevention and intervention programs.

## Data Availability

The datasets presented in this study can be found in online repositories. The names of the repository/repositories and accession number(s) can be found at: Research Data Unipd https://researchdata.cab.unipd.it/id/eprint/1183, DOI: 10.25430/researchdata.cab.unipd.it.00001183.

## References

[ref1] American Psychiatric Association (APA) (2000). Diagnostic and statistical manual of mental disorders: DSM-IV-TR®. Washington, DC: American Psychiatric Association. doi: 10.1176/appi.books.9780890423349

[ref2] Asendorf J. B. Conner M. de Fruyt F. De Houwer J. Denissen J. J. A. Fiedler K.. (2013). Recommendations for increasing replicability in psychology. in European Journal of Personality, 27, 108–119. doi: 10.1002/per.1919

[ref3] Athey R. A. Kershaw V. Radley S. (2021). Systematic review of sexual function in older women. Eur. J. Obstet. Gynecol. Reprod. Biol. 267, 198–204. doi: 10.1016/j.ejogrb.2021.11.011 34826667

[ref4] Bancroft J. (2009). Human sexuality and its problems. Edinburg (EDH): Elsevier Health Sciences.

[ref5] Barone B. Napolitano L. Abate M. Cirillo L. Reccia P. Passaro F.. (2022). The role of testosterone in the elderly: what do we know? Int. J. Mol. Sci. 23:3535. doi: 10.3390/ijms23073535, PMID: 35408895 PMC8998588

[ref6] Basson R. (2000). The female sexual response: A different model. J. Sex Marital Ther. 26, 51–65. doi: 10.1080/009262300278641 10693116

[ref7] Basson R. (2001). Using a different model for female sexual response to address women’s problematic low sexual desire. J. Sex Marital Ther. 27, 395–403. doi: 10.1080/713846827 11554199

[ref8] Baudelot-Berrogain N. Roquejoffre S. Game X. Mallet M. Mouzin N. Bertrand P.. (2006). Linguistic validation of the “brief index of sexual functioning for women”. Application to the study of sexuality in a population of 93 French women. Prog. Urol. 16, 174–183. 16734241

[ref9] Bittoni C. Kiesner J. (2023). When the brain turns on with sexual desire: fMRI findings, issues, and future directions. Sex. Med. Rev. 11, 296–311. doi: 10.1093/sxmrev/qead029 37500582

[ref10] Bjorklund D. F. Kipp K. (1996). Parental investment theory and gender differences in the evolution of inhibition mechanisms. Psychol. Bull. 120, 163–188. doi: 10.1037/0033-2909.120.2.163 8831295

[ref11] Bonechi A. Tani F. (2011). Italian adaptation of the miltidimensional measure of emotional abuse (MMEA). TMP 18, 65–86.

[ref12] Carpenter S. (2018). Ten steps in scale development and reporting: a guide for researchers. Commun. Methods Meas. 12, 25–44. doi: 10.1080/19312458.2017.1396583

[ref13] Chen F. F. (2007). Sensitivity of goodness of fit indexes to lack of measurement invariance. Struct. Equ. Model. 14, 464–504. doi: 10.1080/10705510701301834

[ref14] Cheung G. W. Rensvold R. B. (2002). Evaluating goodness-of-fit indexes for testing measurement invariance. Struct. Equ. Model. 9, 233–255. doi: 10.1207/S15328007SEM0902_5

[ref15] Chivers M. L. Bailey J. M. (2005). A sex difference in features that elicit genital response. Biol. Psychol. 70, 115–120. doi: 10.1016/j.biopsycho.2004.12.002 16168255

[ref16] Dekker A. Schmidt G. (2013). Patterns of masturbatory behaviour: changes between the sixties and the nineties. J. Psychol. Hum. Sex. 14, 35–48.

[ref17] Derogatis L. R. (1997). The derogatis interview for sexual functioning (Disf/disf-sr): an introductory report. J. Sex Marital Ther. 23, 291–304. doi: 10.1080/00926239708403933 9427208

[ref18] Derogatis L. R. (1998). “The Derogatis sexual functioning inventory” in Handbook of sexuality-related measures (pp. 269–271). eds. Fisher T. D. Davis C. M. Yarber W. L. Davis S. L. (New York (NY): Routledge).

[ref19] Dillman D. A. Smyth J. D. Christian L. M. (2014). Internet, phone, mail, and mixed-mode surveys: The tailored design method. Hoboken (NJ): John Wiley & Sons.

[ref20] Fahs B. Swank E. (2021). Reciprocity, partner pressure, and emotional labor: women discuss negotiations around oral and anal sex. Sex. Cult. 25, 217–234. doi: 10.1007/s12119-020-09766-w

[ref21] Farvid P. Braun V. Rowney C. (2017). ‘No girl wants to be called a slut!’: Women, heterosexual casual sex and the sexual double standard. J. Gend. Stud. 26, 544–560. doi: 10.1080/09589236.2016.1150818

[ref22] Ferrucci R. Panzeri M. Ronconi L. Ardolino G. Cogiamanian F. Barbieri S.. (2016). Abnormal sexuality in Parkinson’s disease: fact or fancy? J. Neurol. Sci. 369, 5–10. doi: 10.1016/j.jns.2016.07.058 27653856

[ref23] Filocamo M. T. Serati M. Li Marzi V. Costantini E. Milanesi M. Pietropaolo A.. (2014). The female sexual function index (FSFI): linguistic validation of the Italian version. J. Sex. Med. 11, 447–453. doi: 10.1111/jsm.12389 24224761

[ref24] Fontanesi L. Renaud P. (2014). Sexual presence: toward a model inspired by evolutionary psychology. New Ideas Psychol. 33, 1–7. doi: 10.1016/j.newideapsych.2013.10.001

[ref25] Han Z. Gan Z. Han H. Chen J. Li K. Guan N. (2014). Validity of the Chinese version of the brief index of sexual functioning for women with a new scoring algorithm and comparison of normative and recurrently depressed Han Chinese population. J. Sex. Med. 11, 439–446. doi: 10.1111/jsm.12385 24354391

[ref26] Harlow S. D. Sievert L. L. LaCroix A. Z. Mishra G. D. Woods N. F. (2023). Women’s midlife health: the unfinished research agenda. Women Midlife Health 9:7. doi: 10.1186/s40695-023-00090-5, PMID: 37784201 PMC10546728

[ref27] Harris A. L. Vitzthum V. J. (2013). Darwin's legacy: an evolutionary view of women's reproductive and sexual functioning. J. Sex Res. 50, 207–246. doi: 10.1080/00224499.2012.763085 23480070

[ref28] Hayes R. Dennerstein L. (2005). The impact of aging on sexual function and sexual dysfunction in women: a review of population-based studies. J. Sex. Med. 2, 317–330. doi: 10.1111/j.1743-6109.2005.20356.x 16422862

[ref29] Herbenick D. Fu T. C. Patterson C. (2023). Sexual repertoire, duration of partnered sex, sexual pleasure, and orgasm: findings from a US nationally representative survey of adults. J. Sex Marital Ther. 49, 369–390. doi: 10.1080/0092623X.2022.2126417 36151751

[ref30] Heřmánková B. Šmucrová H. Mikulášová M. Oreská S. Špiritović M. Štorkánová H.. (2021). Validation of Czech versions of questionnaires assessing female sexual function and pelvic floor function. Czech Rheumatol. 29, 30–40.

[ref31] Hu L. T. Bentler P. M. (1999). Cutoff criteria for fit indexes in covariance structure analysis: conventional criteria versus new alternatives. Struct. Equ. Model. Multidiscip. J. 6, 1–55. doi: 10.1080/10705519909540118

[ref32] Huang S. Niu C. Santtila P. (2022). Masturbation frequency and sexual function in individuals with and without sexual partners. Theol. Sex. 3, 229–243. doi: 10.3390/sexes3020018

[ref33] Janssen E. McBride K. R. Yarber W. Hill B. J. Butler S. M. (2008). Factors that influence sexual arousal in men: a focus group study. Arch. Sex. Behav. 37, 252–265. doi: 10.1007/s10508-007-9245-5 18040768

[ref34] Janssen E. Vorst H. Finn P. Bancroft J. (2002). The sexual inhibition (SIS) and sexual excitation (SES) scales: I. Measuring sexual inhibition and excitation proneness in men. J. Sex Res. 39, 114–126. doi: 10.1080/00224490209552130 12476243

[ref35] Johnson S. Zuccarini D. (2010). Integrating sex and attachment in emotionally focused couple therapy. J. Marital. Fam. Ther. 36, 431–445. doi: 10.1111/j.1752-0606.2009.00155.x 21039657

[ref36] Jöreskog K. G. Sörbom D. (1996). LISREL 8: User’s reference guide. Lincolwood, IL: Scientific Software International.

[ref37] Kaplan H. S. (1974). The new sex therapy. Active treatment of sexual dysfunctions. New York (NY): Routledge.

[ref38] Kaplan H. S. (1979). Disorders of sexual desire. New York (NY): Brunner/Mazel.

[ref39] Kline R. B. (2013). “Exploratory and confirmatory factor analysis” in Applied quantitative analysis education and the social sciences. eds. Petscher Y. Schatschneider C. Compton D. L. (New York (NY): Routledge), 171–207.

[ref40] Kok E. L. (2004). Differences between male and female sexual functioning. S. Afr. Fam. Pract. 46, 12–15. doi: 10.1080/20786204.2004.10873065

[ref41] Leavitt C. E. Leonhardt N. D. Busby D. M. Clarke R. W. (2021). When is enough enough? Orgasm’s curvilinear association with relational and sexual satisfaction. J. Sex. Med. 18, 167–178. doi: 10.1016/j.jsxm.2020.10.002 33281078

[ref42] Liddon L. Kingerlee R. Barry J. A. (2018). Gender differences in preferences for psychological treatment, coping strategies, and triggers to help-seeking. Br. J. Clin. Psychol. 57, 42–58. doi: 10.1111/bjc.12147 28691375

[ref43] Mah K. Binik Y. M. (2001). The nature of human orgasm: a critical review of major trends. Clin. Psychol. Rev. 21, 823–856. doi: 10.1016/S0272-7358(00)00069-6 11497209

[ref44] Mah K. Binik Y. M. (2002). Do all orgasms feel alike? Evaluating a two-dimensional model of the orgasm experience across gender and sexual context. J. Sex Res. 39, 104–113. doi: 10.1080/00224490209552129 12476242

[ref45] Masters W. H. Johnson V. E. (1966). Human sexual response. Toronto; New York: Bantam Books.

[ref46] Mazer N. A. Leiblum S. R. Rosen R. C. (2000). The brief index of sexual functioning for women (BISF-W): a new scoring algorithm and comparison of normative and surgically menopausal populations. Menopause 7, 350–363. doi: 10.1097/00042192-200007050-00009 10993034

[ref47] McCabe M. Althof S. E. Assalian P. Chevret-Measson M. Leiblum S. R. Simonelli C.. (2010). Psychological and interpersonal dimensions of sexual function and dysfunction. J. Sex. Med. 7, 327–336. doi: 10.1111/j.1743-6109.2009.01618.x 20092442

[ref48] Gelenberg A. J. Laukes C. A. Manber R. McKnight K. M. Moreno F. A.. (1997). The Arizona sexual experience scale: validity and reliability. In Proceeding of 150th annual meeting of the American Psychiatric Association

[ref49] Norton R. (1983). Measuring marital quality: a critical look at the dependent variable measuring marital quality: a critical look at the dependent variable. J. Marriage Fam. 45, 141–151. doi: 10.2307/351302

[ref50] Panzeri M. (2023). Terapia sessuale mansionale. Un approccio integrato. il Mulino.

[ref51] Panzeri M. Optale G. (2006). “Traduzione e adattamento italiano del brief index of sexual functioning for women (BISF-W)” in VII Congresso Nazionale di Psicologia della Salute. Promuovere benessere con persone gruppi comunità. eds. Cicognani E. Palestrini L. (Cesena (FC): Il Ponte Vecchio), 228–229.

[ref52] Panzeri M. Raoli V. (2010). Il brief index of sexual functioning for men (BISF-M): validazione sulla popolazione Italiana. Rivista Sessuologia Clinica 17, 41–68. doi: 10.3280/RSC2010-001003

[ref53] Panzeri M. Ronconi L. Donà M. A. Optale G. (2009). Il brief index of sexual functioning for women (BISF-W): validazione su un campione italiano. Rivista Sessuologia Clinica 16, 39–62. doi: 10.3280/RSC2009-001002

[ref54] Pappalardo D. Panzeri M. (2015). Sexual arousal and sexual inhibition: qualitative study on Italian men through focus group. J. Sexual Med. 12, 214–241.

[ref55] Pendergast L. L. von der Embse N. Kilgus S. P. Eklund K. R. (2017). Measurement equivalence: a non-technical primer on categorical multi-group confirmatory factor analysis in school psychology. J. Sch. Psychol. 60, 65–82. doi: 10.1016/j.jsp.2016.11.002 28164800

[ref56] Pickles J. Hirst J. Froggatt C. Kenny M. (2023). Perceptions of young women who engage in anal sex: a sociological inquiry. J. Posit. Sexual. 9, 14–21. doi: 10.51681/1.913

[ref57] Pinkerton S. D. Bogart L. M. Cecil H. Abramson P. R. (2003). Factors associated with masturbation in a collegiate sample. J. Psychol. Hum. Sex. 14, 103–121. doi: 10.1300/J056v14n02_07

[ref58] Pinxten W. Lievens J. (2014). An exploratory study of factors associated with sexual inhibition and excitation: findings from a representative survey in Flanders. J. Sex Res. 52, 679–689. doi: 10.1080/00224499.2014.882880 24670220

[ref59] Reynolds G. L. Fisher D. G. Rogala B. (2015). Why women engage in anal intercourse: results from a qualitative study. Arch. Sex. Behav. 44, 983–995. doi: 10.1007/s10508-014-0367-2, PMID: 25378264 PMC4379393

[ref61] Rosen R. Brown C. Heiman J. Leiblum S. Meston C. Shabsigh R.. (2000). The female sexual function index (FSFI): a multidimensional self-report instrument for the assessment of female sexual function The fem ale sexual function index (FSFI): a multidimension al self-report Instrument for the Assessment of femal. J Sex Marital Th. 26, 191–208. doi: 10.1080/009262300278597 10782451

[ref62] Rosen R. Riley A. Wagner G. Osterloh I. H. Kirkpatrick J. Mishra A. (1997). The international index of erectile function (IIEF): a multidimensional scale for assessment of erectile dysfunction. Urology 49, 822–830. doi: 10.1016/S0090-4295(97)00238-0 9187685

[ref63] Shackelford T. K. Goetz A. T. (2007). Adaptation to sperm competition in humans. Curr. Dir. Psychol. Sci. 16, 47–50. doi: 10.1111/j.1467-8721.2007.00473.x

[ref64] Spector I. P. Carey M. P. Steinberg L. (1996). The sexual desire inventory: development, factor structure, and evidence of reliability. J. Sex Marital Ther. 22, 175–190. doi: 10.1080/00926239608414655 8880651

[ref65] Štulhofer A. Jurin T. Graham C. Janssen E. Træen B. (2019). Emotional intimacy and sexual well-being in aging European couples: a cross-cultural mediation analysis. Eur. J. Ageing 17, 43–54. doi: 10.1007/s10433-019-00509-x, PMID: 32158371 PMC7040139

[ref66] Syme M. L. Cohn T. J. Stoffregen S. Kaempfe H. Schippers D. (2018). “At my age …”: defining sexual wellness in mid- and later life. J. Sex Res. 56, 832–842. doi: 10.1080/00224499.2018.1456510 29668312

[ref67] Symons D. (1980). The evolution of human-sexuality. Behavioral and Brain Sciences, 3. doi: 10.1017/S0140525X0000412X

[ref68] Taber K. S. (2018). The use of Cronbach’s alpha when developing and reporting research instruments in science education. Res. Sci. Educ. 48, 1273–1296. doi: 10.1007/s11165-016-9602-2

[ref69] Tavares I. M. Moura C. V. Nobre P. J. (2020). The role of cognitive processing factors in sexual function and dysfunction in women and men: a systematic review. Sex. Med. Rev. 8, 403–430. doi: 10.1016/j.sxmr.2020.03.002 32402763

[ref70] Taylor J. F. Rosen R. C. Leiblum S. R. (1994). Self-report assessment of female sexual function: psychometric evaluation of the brief index of sexual functioning for women. Arch. Sex. Behav. 23, 627–643. doi: 10.1007/BF01541816 7872859

[ref71] Wiegel M. Meston C. Rosen R. (2005). The female sexual function index (FSFI): cross-validation and development of clinical cutoff scores. J. Sex Marital Ther. 31, 1–20. doi: 10.1080/00926230590475206 15841702

[ref72] Witherow M. P. Chandraiah S. Seals S. R. Sarver D. E. Parisi K. E. Bugan A. (2017). Relational intimacy mediates sexual outcomes associated with impaired sexual function: examination in a clinical sample. J. Sex. Med. 14, 843–851. doi: 10.1016/j.jsxm.2017.04.671 28583343

[ref73] Zemishlany Z. Weizman A. (2008). The impact of mental illness on sexual dysfunction. Sex. Dysfunct. 29, 89–106. doi: 10.1159/000126626 18391559

