# Supplementary material for: Validation of the Brief Index of Sexual Functioning for women and men (BISF-W and BISF-M) in an Italian sample
Source: Front Psychol. 2024 Nov 6;15:1474288. doi: 10.3389/fpsyg.2024.1474288 (PMC11576204; doi:10.3389/fpsyg.2024.1474288)
Supplement: Supplementary file 1 [file Table_1.DOCX]

| **S2.1. Factor loadings of 4-factors model - Women** | | | | |
| --- | --- | --- | --- | --- |
|  |  |  |  |  |
| Item | Factor loadings |  | Item | Factor loadings |
| Factor -1 | |  | q17 | ,63 |
| q4_1 | ,48 |  | q20 | ,39 |
| q4_4 | ,63 |  |  |  |
| q4_5 | ,52 |  | Factor-2 | |
| q4_6 | ,68 |  | q3 | ,72 |
| q5_1 | ,64 |  | q4_2 | ,46 |
| q5_4 | ,61 |  | q5_2 | ,78 |
| q5_5 | ,80 |  | q5_3 | ,45 |
| q5_6 | ,66 |  | q7_2 | ,88 |
| q5_7 | ,84 |  | q7_3 | ,47 |
| q7_1 | ,59 |  | q11_3 | ,42 |
| q7_4 | ,62 |  |  |  |
| q7_5 | ,78 |  | Factor-3 |  |
| q7_6 | ,64 |  | q6 | ,45 |
| q7_7 | ,76 |  | q13_5 | ,23 |
| q9 | ,78 |  | q14_2 | ,29 |
| q10 | ,83 |  | q14_3 | ,28 |
| q11_2 | ,34 |  | q14_4 | ,30 |
| q11_4 | ,61 |  | q14_5 | ,27 |
| q11_5 | ,59 |  | q18 | ,82 |
| q11_6 | ,61 |  | q19 | ,82 |
| q11_7 | ,78 |  |  |  |
| q12 | ,35 |  | Factor-4 |  |
| q13_1 | ,53 |  | q4_7 | ,79 |
| q13_2 | ,63 |  | q5_8 | ,93 |
| q13_3 | ,70 |  | q7_8 | ,86 |
| q13_4 | ,74 |  | q11_8 | ,84 |

| **S2.2. Factor loadings of 4-factors model - Men** | | | |  |
| --- | --- | --- | --- | --- |
|  |  |  |  |  |
| Item | Factor loadings |  | Item | Factor loadings |
| Factor -1 | |  | q17 | ,71 |
| q4_1 | ,39 |  | q20 | ,37 |
| q4_4 | ,49 |  |  |  |
| q4_5 | ,38 |  | Factor-2 | |
| q4_6 | ,49 |  | q3 | ,70 |
| q5_1 | ,62 |  | q4_2 | ,56 |
| q5_4 | ,60 |  | q5_2 | ,70 |
| q5_5 | ,77 |  | q5_3 | ,55 |
| q5_6 | ,70 |  | q7_2 | ,81 |
| q5_7 | ,81 |  | q7_3 | ,57 |
| q7_1 | ,70 |  | q11_3 | ,51 |
| q7_4 | ,62 |  |  |  |
| q7_5 | ,78 |  | Factor-3 |  |
| q7_6 | ,64 |  | q6 | ,30 |
| q7_7 | ,74 |  | q13_5 | ,19 |
| q9 | ,77 |  | q14_2 | ,18 |
| q10 | ,80 |  | q14_3 | ,14 |
| q11_2 | ,15 |  | q14_4 | ,15 |
| q11_4 | ,56 |  | q14_0 | ,14 |
| q11_5 | ,46 |  | q18 | ,76 |
| q11_6 | ,62 |  | q19 | ,90 |
| q11_7 | ,79 |  |  |  |
| q12 | ,31 |  | Factor-4 |  |
| q13_1 | ,31 |  | q4_7 | ,66 |
| q13_2 | ,43 |  | q5_8 | ,96 |
| q13_3 | ,58 |  | q7_8 | ,85 |
| q13_4 | ,63 |  | q11_8 | ,93 |
