# Supplementary material for: Validation of the Brief Index of Sexual Functioning for women and men (BISF-W and BISF-M) in an Italian sample
Source: Front Psychol. 2024 Nov 6;15:1474288. doi: 10.3389/fpsyg.2024.1474288 (PMC11576204; doi:10.3389/fpsyg.2024.1474288)
Supplement: Supplementary file 2 [file Table_2.DOCX]

**S1.1 The BISF-W (Italian version; Panzeri et al. 2009; English version in Taylor et al. 1994)**

|  | Dr. Marta Panzeri  Università degli Studi di Padova  DPSS / Dipartimento di Psicologia  dello Sviluppo e della Socializzazione  via Venezia, 8 - 35131 PADOVA (ITALY) - tel. 049 8278468  e-mail: marta.panzeri@unipd.it |
| --- | --- |

Cerchi di leggere le domande con molta attenzione, facendo un segno sulla casella che più corrisponde alla Sua realtà.

Tenga presente che non ci sono risposte giuste o sbagliate.

Di seguito vengono riportate le definizioni di alcuni termini specifici presenti nel questionario.

Masturbazione: stimolazione dei genitali al fine di procurarsi piacere sessuale

Coito: rapporto sessuale con introduzione del pene in vagina

Sesso orale: rapporto sessuale che consiste nella stimolazione orale (cioè con la bocca) del pene o dei genitali femminili

Sesso anale: rapporto sessuale con l’introduzione del pene nell’ano

Lubrificazione vaginale: sensazione di essere bagnate nella zona genitale provata durante l’eccitazione sessuale

Orgasmo: massima sensazione breve ed intensa del piacere sessuale, caratterizzato da contrazioni ritmiche dell’area genitale

Costrizione vaginale: sensazione che la vagina sia chiusa o troppo stretta

Risponda alle seguenti domande scegliendo la risposta più precisa *per quanto riguarda* ***l’ultimo mese****.*

1. Nell’ultimo mese ha avuto un partner sessuale fisso? Sì **□** No **□**

Da quanto tempo sta con il suo partner attuale (specificare mesi e anni)?__________________

2. Nell’ultimo mese ha avuto dei partner occasionali? Sì **□** No **□**

3. Durante l’ultimo mese, quante volte ha avuto fantasie o pensieri sessuali, o sogni erotici? *(Si prega di segnare la risposta più esatta.)*

0 Mai

1. Una volta
2. 2 o 3 volte
3. Una volta alla settimana
4. 2 o 3 volte alla settimana
5. Una volta al giorno
6. Più spesso

4. Utilizzando la scala numerica riportata qui sotto, indichi quante volte durante l’ultimo mese ha sentito il desiderio di partecipare alle seguenti attività. *(Una risposta è necessaria per ciascuna, anche se non Le è pertinente)*

|  | **0 *=*** Mai | **1 *=*** Una volta | **2 *=*** 2 o 3 volte | **3 *=*** Una volta alla settimana | **4 *=*** *2* o 3 volte alla settimana | **5 =** Una volta al giorno | **6 =** Più spesso |
| --- | --- | --- | --- | --- | --- | --- | --- |
| 4_1 Baciare | 0 | 1 | 2 | 3 | 4 | 5 | 6 |
| 4_2 Masturbazione da sola | 0 | 1 | 2 | 3 | 4 | 5 | 6 |
| 4_3 Masturbazione reciproca | 0 | 1 | 2 | 3 | 4 | 5 | 6 |
| 4_4 Accarezzare e giochi preliminari | 0 | 1 | 2 | 3 | 4 | 5 | 6 |
| 4_5 Sesso orale | 0 | 1 | 2 | 3 | 4 | 5 | 6 |
| 4_6 Penetrazione vaginale o coito | 0 | 1 | 2 | 3 | 4 | 5 | 6 |
| 4_7 Sesso anale | 0 | 1 | 2 | 3 | 4 | 5 | 6 |

5. Utilizzando la scala numerica riportata qui sotto, indichi quante volte durante l’ultimo mese si è sentito eccitato nello svolgere le seguenti attività. *(Una risposta è necessaria per ciascuna, anche se non Le è pertinente)*

|  | **0 *=*** Non ho praticato questa attività | **1 *=*** Per niente | **2 *=*** raramente, in meno del 25% delle occasioni | **3 *=*** Circa metà delle occasio-ni | **4 *=*** Di solito, circa il 75% delle occasioni | **5 *=*** Mi eccito sempre |
| --- | --- | --- | --- | --- | --- | --- |
| 5_1 Baciare | 0 | 1 | 2 | 3 | 4 | 5 |
| 5_2 Sogni o fantasie | 0 | 1 | 2 | 3 | 4 | 5 |
| 5_3 Masturbazione da sola | 0 | 1 | 2 | 3 | 4 | 5 |
| 5_4 Masturbazione reciproca | 0 | 1 | 2 | 3 | 4 | 5 |
| 5_5 Accarezzare e giochi preliminari | 0 | 1 | 2 | 3 | 4 | 5 |
| 5_6 Sesso orale | 0 | 1 | 2 | 3 | 4 | 5 |
| 5_7 Penetrazione vaginale o coito | 0 | 1 | 2 | 3 | 4 | 5 |
| 5_8 Sesso anale | 0 | 1 | 2 | 3 | 4 | 5 |

6. Complessivamente, nell’ultimo mese, quanto frequentemente si è sentita apprensiva o inibita durante l’attività sessuale con un partner? *(Si prega di segnare la risposta più esatta.)*

1. Non ho avuto un partner in questo periodo
2. Per niente ansiosa o inibita

2 Raramente, in meno del 25% delle occasioni

3. Circa metà delle occasioni

4 Di solito, circa 75% delle occasioni

1. Sempre divento ansiosa o inibita

7. Utilizzando la scala numerica riportata qui sotto, indichi quante volte durante l’ultimo mese ha partecipato alle seguenti esperienze sessuali. *(Una risposta è necessaria per ciascuna, anche se non Le è pertinente)*

|  | **0 *=*** Mai | **1 *=*** Una volta | **2 *=*** 2 o 3 volte | **3 *=*** Una volta alla settimana | **4 *=*** *2* o 3 volte alla settimana | **5 =** Una volta al giorno | **6 =** Più spesso |
| --- | --- | --- | --- | --- | --- | --- | --- |
| 7_1 Baciare | 0 | 1 | 2 | 3 | 4 | 5 | 6 |
| 7_2 Fantasie sessuali | 0 | 1 | 2 | 3 | 4 | 5 | 6 |
| 7_3 Masturbazione da sola | 0 | 1 | 2 | 3 | 4 | 5 | 6 |
| 7_4 Masturbazione reciproca | 0 | 1 | 2 | 3 | 4 | 5 | 6 |
| 7_5 Accarezzare e giochi preliminari | 0 | 1 | 2 | 3 | 4 | 5 | 6 |
| 7_6 Sesso orale | 0 | 1 | 2 | 3 | 4 | 5 | 6 |
| 7_7 Penetrazione vaginale o coito | 0 | 1 | 2 | 3 | 4 | 5 | 6 |
| 7_8 Sesso anale | 0 | 1 | 2 | 3 | 4 | 5 | 6 |

8. Durante l’ultimo mese, di solito chi iniziava l’attività sessuale? *(Si prega di segnare la risposta più esatta.)*

1. Non ho avuto un partner in questo periodo
2. Non ho avuto rapporti con un partner nell’ultimo mese
3. Di solito iniziavo io l’attività
4. Iniziamo in uguale misura io e il mio partner l’attività
5. Di solito inizia il mio partner l’attività

9 Nell’ultimo mese, di solito come reagiva alle *avance* del Suo partner? *(Si prega di segnare la risposta più esatta.)*

1. Non ho avuto un partner in questo periodo
2. Non è successo nell’ultimo mese
3. Di solito rifiutavo
4. A volte rifiutavo
5. Accettavo controvoglia
6. Accettavo, ma non necessariamente con piacere
7. Di solito accettavo con piacere
8. Accettavo sempre con piacere

10. Nell’ultimo mese, ha provato piacere da qualsiasi forma di esperienza sessuale? *(Si prega di segnare la risposta più esatta.)*

1. Non ho avuto un partner in questo periodo
2. Non ho avuto esperienze sessuali nell’ultimo mese
3. Non ho provato nessun piacere

3. Raramente, in meno del 25% delle occasioni

4. Circa metà delle occasioni

5. Di solito, circa 75% delle occasioni

1. Ho sempre provato piacere

11. Utilizzando la scala numerica riportata qui sotto, indichi quanto spesso durante l’ultimo mese ha raggiunto l’orgasmo con le seguenti attività. *(Una risposta è necessaria per ciascuna, anche se non Le è pertinente)*

|  | **0 *=*** Non ho praticato questa attività | **1 *=*** Per niente | **2 *=*** raramente, in meno del 25% delle occasioni | **3 *=*** Circa metà delle occasio-ni | **4 *=*** Di solito, circa il 75% delle occasioni | **5 *=*** Ho sempre raggiunto l’orgasmo |
| --- | --- | --- | --- | --- | --- | --- |
| 11_1 Baciare | 0 | 1 | 2 | 3 | 4 | 5 |
| 11_2 Sogni o fantasie | 0 | 1 | 2 | 3 | 4 | 5 |
| 11_3 Masturbazione da sola | 0 | 1 | 2 | 3 | 4 | 5 |
| 11_4 Masturbazione reciproca | 0 | 1 | 2 | 3 | 4 | 5 |
| 11_5 Accarezzare e giochi preliminari | 0 | 1 | 2 | 3 | 4 | 5 |
| 11_6 Sesso orale | 0 | 1 | 2 | 3 | 4 | 5 |
| 11_7 Penetrazione vaginale o coito | 0 | 1 | 2 | 3 | 4 | 5 |
| 11_8 Sesso anale | 0 | 1 | 2 | 3 | 4 | 5 |

12. Durante l’ultimo mese, la frequenza della Sua attività con un partner è stata:

*(Si prega di segnare la risposta più esatta.)*

1. Non ho avuto un partner in questo periodo
2. Meno di quanto Lei desiderasse
3. Tanto quanto Lei desiderasse
4. Più di quanto Lei desiderasse

13. Utilizzando la scala numerica riportata qui sotto, indichi ogni eventuale livello di cambiamento nelle seguenti categorie per l’ultimo mese. *(Una risposta è necessaria per ciascuna categoria, anche se non Le è pertinente)*

|  | **0 *=*** Non applicabile | **1 *=*** Molto più basso | **2 *=*** Un po’ più basso | **3 *=*** Nessun cambiamento | **4 *=*** Un po’ più alto | **5 *=*** Molto più alto |
| --- | --- | --- | --- | --- | --- | --- |
| 13_1 Interesse sessuale | 0 | 1 | 2 | 3 | 4 | 5 |
| 13_2 Eccitamento sessuale | 0 | 1 | 2 | 3 | 4 | 5 |
| 13_3 Attività sessuale | 0 | 1 | 2 | 3 | 4 | 5 |
| 13_4 Soddisfazione sessuale | 0 | 1 | 2 | 3 | 4 | 5 |
| 13_5 Apprensione sessuale | 0 | 1 | 2 | 3 | 4 | 5 |

14. Utilizzando la scala numerica riportata qui sotto, indichi quanto durante l’ultimo mese ha provato le seguenti condizioni? *(Una risposta è necessaria per ciascuna, anche se non Le è pertinente.)*

|  | **0 *=*** Per niente | **1 *=*** raramente, in meno del 25% delle occasioni | **2 *=*** Circa metà delle occasioni | **3 *=*** Di solito, circa il 75% delle occasioni | **4 *=*** Sempre |
| --- | --- | --- | --- | --- | --- |
| 14_1 Sanguinamento o irritazione in seguito alla penetrazione vaginale o coito | 0 | 1 | 2 | 3 | 4 |
| 14_2 Scarsa lubrificazione vaginale | 0 | 1 | 2 | 3 | 4 |
| 14_3 Penetrazione o coito doloroso | 0 | 1 | 2 | 3 | 4 |
| 14_4 Difficoltà nel raggiungere l’orgasmo | 0 | 1 | 2 | 3 | 4 |
| 14_5 Costrizione vaginale | 0 | 1 | 2 | 3 | 4 |
| 14_6 Orinazione involontaria | 0 | 1 | 2 | 3 | 4 |
| 14_7 Mal di testa dopo l’attività sessuale | 0 | 1 | 2 | 3 | 4 |
| 14_8 Infezione vaginale | 0 | 1 | 2 | 3 | 4 |

15. Utilizzando la scala numerica riportata qui sotto, indichi quanto hanno influito sul Suo livello di attività sessuale i seguenti fattori durante l’ultimo mese. *(Una risposta è necessaria per ciascuna, anche se non Le è pertinente)*

|  | **0 *=*** Per niente | **1 *=*** raramente,  in meno del 25% delle occasioni | **2 *=*** Circa metà delle occasio-ni | **3 *=*** Di solito, circa il 75% delle occasioni | **4 *=*** Sempre |
| --- | --- | --- | --- | --- | --- |
| 15_1 Problemi con la mia salute (p.e., infezioni, malattie) | 0 | 1 | 2 | 3 | 4 |
| 15_2 La salute del/la mio/a partner | 0 | 1 | 2 | 3 | 4 |
| 15_3 Conflitti nel nostro rapporto | 0 | 1 | 2 | 3 | 4 |
| 15_4 Mancanza di *privacy* | 0 | 1 | 2 | 3 | 4 |
| 15_5 Altro *(si prega di specificare)* | 0 | 1 | 2 | 3 | 4 |

16. Quanto è soddisfatta dell’aspetto complessivo del Suo corpo? *(Si prega di segnare la risposta più esatta.)*

1. Molto soddisfatta
2. Abbastanza soddisfatta
3. Né soddisfatta né insoddisfatta
4. Abbastanza insoddisfatta
5. Molto insoddisfatta.

17. Nell’ultimo mese, quanto frequentemente è riuscita a comunicare al partner i Suoi desideri o preferenze sessuali? *(Si prega di segnare la risposta più esatta.)*

0. Non ho avuto un partner in questo periodo

1. Non sono riuscita a comunicare i miei desideri e preferenze

2. Raramente, in meno del 25% delle occasioni

3. Circa metà delle occasioni

4. Di solito, circa 75%

5. Sono sempre riuscita a comunicare i miei desideri e preferenze.

18. Complessivamente, quanto è soddisfatta del rapporto sessuale col Suo partner? *(Si prega di segnare la risposta più esatta.)*

1. Non ho avuto un partner in questo periodo
2. Molto soddisfatta
3. Abbastanza soddisfatta
4. Né soddisfatta né insoddisfatta
5. Abbastanza insoddisfatta
6. Molto insoddisfatta

19. Complessivamente, quanto crede che sia soddisfatto del Vostro rapporto sessuale il Suo partner? *(Si prega di segnare la risposta più esatta.)*

1. Non ho avuto un partner in questo periodo
2. Molto soddisfatto
3. Abbastanza soddisfatto
4. Né soddisfatto né insoddisfatto
5. Abbastanza insoddisfatto
6. Molto insoddisfatto

20. Complessivamente, quanto Le importa nella vita la Sua attività sessuale? *(Si prega di segnare la risposta più esatta.)*

1. Non è affatto importante
2. Non molto importante
3. Né importante né poco importante
4. Abbastanza importante
5. Molto importante.

21. Indichi il numero che corrisponde alla frase che meglio descrive la Sua esperienza sessuale. (eterosessuale: che ha rapporti con persone dell’altro sesso; omosessuale: che ha rapporti con persone dello stesso sesso).

1. Interamente eterosessuale
2. Principalmente eterosessuale, con qualche esperienza omosessuale
3. Principalmente eterosessuale, ma con considerevole esperienza omosessuale
4. Ugualmente eterosessuale e omosessuale
5. Principalmente omosessuale, ma con considerevole esperienza eterosessuale
6. Principalmente omosessuale, con qualche esperienza eterosessuale
7. Completamente omosessuale

22. Indichi il numero che corrisponde alla frase che meglio descrive i Suoi desideri sessuali.

1. Interamente eterosessuale
2. Principalmente eterosessuale, con qualche desiderio omosessuale
3. Principalmente eterosessuale, ma con considerevole desiderio omosessuale
4. Ugualmente eterosessuale e omosessuale
5. Principalmente omosessuale, ma con considerevole desiderio eterosessuale
6. Principalmente omosessuale, con qualche desiderio eterosessuale
7. Completamente omosessuale.

**S1.2 BISF-W factors composition**

COMPUTE Dyadic = MEAN(Q10,Q5_7,Q11_7,Q7_5,Q7_7,Q9,Q5_5,Q13_4,Q13_3,Q17,Q13_2,Q5_1,Q7_1,Q12,Q11_5,Q4_4,Q4_6,Q7_6,Q5_6,Q13_1,Q7_4,

Q11_6, Q4_1,Q11_4,Q5_4,Q20,Q11_2) .

VARIABLE LABELS Dyadic 'Dyadic Sexuality’ .

EXECUTE .

COMPUTE Solit = MEAN(Q4_2,Q7_3,Q5_3,q11_3,Q5_2,Q3,Q7_2) .

VARIABLE LABELS Solit 'Solitary Sexuality' .

EXECUTE .

COMPUTE Difficulties = MEAN(Q14_3,Q14_2,Q19,Q6,Q14_4,Q18,Q14_5,Q13_5) .

VARIABLE LABELS Difficulties 'Sexual Difficulties’ .

EXECUTE .

COMPUTE Anal = MEAN(Q5_8,Q7_8,q11_8,Q4_7,Q4_5) .

VARIABLE LABELS Anal 'Anal Sexuality’ .

EXECUTE .

**S1.3 The BISF-M (Panzeri & Raoli 2010)**

|  | Dr. Marta Panzeri  Università degli Studi di Padova  DPSS / Dipartimento di Psicologia  dello Sviluppo e della Socializzazione  via Belzoni, 80 - 35131 PADOVA (ITALY) - Tel. 049 8278467 - fax 049-8278451  e-mail: marta.panzeri@unipd.it |
| --- | --- |

Cerchi di leggere le domande con molta attenzione, facendo un segno sulla casella che più corrisponde alla Sua realtà.

Tenga presente che non ci sono risposte giuste o sbagliate.

Di seguito vengono riportate le definizioni di alcuni termini specifici presenti nel questionario.

Masturbazione: manipolazione dei genitali al fine di procurarsi piacere sessuale

Coito: rapporto sessuale con introduzione del pene in vagina

Sesso orale: rapporto sessuale che consiste nella stimolazione orale (cioè con la bocca) del pene o dei genitali femminili

Sesso anale: rapporto sessuale con l’introduzione del pene nell’ano

Erezione: aumento di volume e rigidità del pene

Eiaculazione: emissione dello sperma al culmine dell’orgasmo

Risponda alle seguenti domande scegliendo la risposta più precisa *per quanto riguarda* ***l’ultimo mese****.*

Q1. Nell’ultimo mese ha avuto un/a partner sessuale fisso/a? Sì **□** No **□**

Da quanto tempo sta con il/la suo/a partner sessuale attuale (specificare mesi e anni)? ______________

Q2. Nell’ultimo mese ha avuto dei partner sessuali occasionali? Sì **□** No **□**

Q3. Durante l’ultimo mese, quante volte ha avuto fantasie o pensieri sessuali o sogni erotici? *(Si prega di segnare la risposta più esatta)*

0. Mai

1. Una volta

1. 2 o 3 volte
2. Una volta alla settimana
3. 2 o 3 volte alla settimana
4. Una volta al giorno
5. Più spesso

Q4. Utilizzando la scala numerica riportata qui sotto, indichi quante volte durante l’ultimo mese ha sentito il desiderio di partecipare alle seguenti attività. *(Una risposta è necessaria per ciascuna, anche se non Le è pertinente)*

|  | **0 *=*** Mai | **1 *=*** Una volta | **2 *=*** 2 o 3 volte | **3 *=*** Una volta alla settimana | **4 *=*** *2* o 3 volte alla settimana | **5 =** Una volta al giorno | **6 =** Più spesso |
| --- | --- | --- | --- | --- | --- | --- | --- |
| 4_1 Baciare | 0 | 1 | 2 | 3 | 4 | 5 | 6 |
| 4_2 Masturbazione da solo | 0 | 1 | 2 | 3 | 4 | 5 | 6 |
| 4_3 Masturbazione reciproca | 0 | 1 | 2 | 3 | 4 | 5 | 6 |
| 4_4 Accarezzare e giochi preliminari | 0 | 1 | 2 | 3 | 4 | 5 | 6 |
| 4_5 Sesso orale | 0 | 1 | 2 | 3 | 4 | 5 | 6 |
| 4_6 Penetrazione vaginale o coito | 0 | 1 | 2 | 3 | 4 | 5 | 6 |
| 4_7 Sesso anale | 0 | 1 | 2 | 3 | 4 | 5 | 6 |

5. Utilizzando la scala numerica riportata qui sotto, indichi quante volte durante l’ultimo mese si è sentito eccitato nello svolgere le seguenti attività. *(Una risposta è necessaria per ciascuna, anche se non Le è pertinente)*

|  | **0 *=*** Non ho praticato questa attività | **1 *=*** Per niente | **2 *=*** raramente, in meno del 25% delle occasioni | **3 *=*** Circa metà delle occasio-ni | **4 *=*** Di solito, circa il 75% delle occasioni | **5 *=*** Mi eccito sempre |
| --- | --- | --- | --- | --- | --- | --- |
| 5_1 Baciare | 0 | 1 | 2 | 3 | 4 | 5 |
| 5_2 Sogni o fantasie | 0 | 1 | 2 | 3 | 4 | 5 |
| 5_2 Masturbazione da solo | 0 | 1 | 2 | 3 | 4 | 5 |
| 5_3 Masturbazione reciproca | 0 | 1 | 2 | 3 | 4 | 5 |
| 5_ 4 Accarezzare e giochi preliminari | 0 | 1 | 2 | 3 | 4 | 5 |
| 5_5 Sesso orale | 0 | 1 | 2 | 3 | 4 | 5 |
| 5_6 Penetrazione vaginale o coito | 0 | 1 | 2 | 3 | 4 | 5 |
| 5_7 Sesso anale | 0 | 1 | 2 | 3 | 4 | 5 |

6. Complessivamente, nell’ultimo mese, quanto frequentemente si è sentito in ansia durante l’attività sessuale con un/a partner per la paura di un insuccesso? *(Si prega di segnare la risposta più esatta)*

1. Non ho avuto un/a partner in questo periodo
2. Per niente ansioso
3. Raramente, in meno del 25% delle occasioni
4. Circa metà delle occasioni
5. Di solito, circa 75% delle occasioni
6. Sempre divento ansioso

7. Utilizzando la scala numerica riportata qui sotto, indichi quante volte durante l’ultimo mese ha partecipato alle seguenti esperienze sessuali. *(Una risposta è necessaria per ciascuna, anche se non Le è pertinente)*

|  | **0 *=*** Mai | **1 *=*** Una volta | **2 *=*** 2 o 3 volte | **3 *=*** Una volta alla settimana | **4 *=*** *2* o 3 volte alla settimana | **5 =** Una volta al giorno | **6 =** Più spesso |
| --- | --- | --- | --- | --- | --- | --- | --- |
| 7_1 Baciare | 0 | 1 | 2 | 3 | 4 | 5 | 6 |
| 7_2 Fantasie sessuali | 0 | 1 | 2 | 3 | 4 | 5 | 6 |
| 7_ 3 Masturbazione da solo | 0 | 1 | 2 | 3 | 4 | 5 | 6 |
| 7_4 Masturbazione reciproca | 0 | 1 | 2 | 3 | 4 | 5 | 6 |
| 7_ 5 Accarezzare e giochi preliminari | 0 | 1 | 2 | 3 | 4 | 5 | 6 |
| 7_ 6 Sesso orale | 0 | 1 | 2 | 3 | 4 | 5 | 6 |
| 7_7 Penetrazione vaginale o coito | 0 | 1 | 2 | 3 | 4 | 5 | 6 |
| 7_8 Sesso anale | 0 | 1 | 2 | 3 | 4 | 5 | 6 |

8. Durante l’ultimo mese, di solito chi iniziava l’attività sessuale? *(Si prega di segnare la risposta più esatta)*

- 1. Non ho avuto un/a partner in questo periodo
  2. Non ho avuto rapporti con un/a partner nell’ultimo mese
  3. Di solito iniziavo io l’attività
  4. Iniziamo in uguale misura io e il/la mio/a partner l’attività
  5. Di solito inizia il/la mio/a partner l’attività

9 Nell’ultimo mese, di solito come reagiva alle *avance* del/la Suo/a partner? *(Si prega di segnare la risposta più esatta)*

1. Non ho avuto un/a partner in questo periodo
2. Non è successo nell’ultimo mese
3. Di solito rifiutavo
4. A volte rifiutavo
5. Accettavo controvoglia
6. Accettavo, ma non necessariamente con piacere
7. Di solito accettavo con piacere
8. Accettavo sempre con piacere

10. Nell’ultimo mese, ha provato piacere da qualsiasi forma di esperienza sessuale? *(Si prega di segnare la risposta più esatta)*

0. Non ho avuto un/a partner in questo periodo

1. Non ho avuto esperienze sessuali nell’ultimo mese

1. Non ho provato nessun piacere

3. Raramente, in meno del 25% delle occasioni

4. Circa metà delle occasioni

5. Di solito, circa 75% delle occasioni

6. Ho sempre provato piacere

11. Utilizzando la scala numerica riportata qui sotto, indichi quanto spesso durante l’ultimo mese ha raggiunto l’orgasmo con le seguenti attività. *(Una risposta è necessaria per ciascuna, anche se non Le è pertinente)*

|  | **0 *=*** Non ho praticato questa attività | **1 *=*** Per niente | **2 *=*** raramente, in meno del 25% delle occasioni | **3 *=*** Circa metà delle occasio-ni | **4 *=*** Di solito, circa il 75% delle occasioni | **5 *=*** Ho sempre raggiunto l’orgasmo |
| --- | --- | --- | --- | --- | --- | --- |
| 11_1 Baciare | 0 | 1 | 2 | 3 | 4 | 5 |
| 11_2 Sogni o fantasie | 0 | 1 | 2 | 3 | 4 | 5 |
| 11_3 Masturbazione da solo | 0 | 1 | 2 | 3 | 4 | 5 |
| 11_4 Masturbazione reciproca | 0 | 1 | 2 | 3 | 4 | 5 |
| 11_ 5 Accarezzare e giochi preliminari | 0 | 1 | 2 | 3 | 4 | 5 |
| 11_ 6 Sesso orale | 0 | 1 | 2 | 3 | 4 | 5 |
| 11_ 7 Penetrazione vaginale o coito | 0 | 1 | 2 | 3 | 4 | 5 |
| 11_ 8 Sesso anale | 0 | 1 | 2 | 3 | 4 | 5 |

12. Durante l’ultimo mese, la frequenza della Sua attività con un/a partner è stata:

*(Si prega di segnare la risposta più esatta)*

1. Non ho avuto un/a partner in questo periodo
2. Meno di quanto Lei desiderasse
3. Tanto quanto Lei desiderasse
4. Più di quanto Lei desiderasse

13. Utilizzando la scala numerica riportata qui sotto, indichi ogni eventuale livello di cambiamento nelle seguenti categorie per l’ultimo mese. *(Una risposta è necessaria per ciascuna categoria, anche se non Le è pertinente)*

|  | **0 *=*** Non applicabile | **1 *=*** Molto più basso | **2 *=*** Un po’ più basso | **3 *=*** Nessun cambiamento | **4 *=*** Un po’ più alto | **5 *=*** Molto più alto |
| --- | --- | --- | --- | --- | --- | --- |
| 13_1 Interesse sessuale | 0 | 1 | 2 | 3 | 4 | 5 |
| 13_2 Eccitazione sessuale | 0 | 1 | 2 | 3 | 4 | 5 |
| 13_3 Attività sessuale | 0 | 1 | 2 | 3 | 4 | 5 |
| 13_4 Soddisfazione sessuale | 0 | 1 | 2 | 3 | 4 | 5 |
| 13_5 Apprensione sessuale | 0 | 1 | 2 | 3 | 4 | 5 |

14. Utilizzando la scala numerica riportata qui sotto, indichi quanto durante l’ultimo mese ha provato le seguenti condizioni? *(Una risposta è necessaria per ciascuna, anche se non Le è pertinente)*

|  | **0 *=*** Per niente | **1 *=*** raramente, in meno del 25% delle occasioni | **2 *=*** Circa metà delle occasioni | **3 *=*** Di solito, circa il 75% delle occasioni | **4 *=*** Sempre |
| --- | --- | --- | --- | --- | --- |
| 14_0 Eiaculazione raggiunta troppo presto | 0 | 1 | 2 | 3 | 4 |
| 14_2 Difficoltà a raggiungere o mantenere l’erezione | 0 | 1 | 2 | 3 | 4 |
| 14_3 Penetrazione o coito doloroso | 0 | 1 | 2 | 3 | 4 |
| 14_4 Eiaculazione non raggiunta o raggiunta con difficoltà | 0 | 1 | 2 | 3 | 4 |
| 14_ 6 Orinazione involontaria | 0 | 1 | 2 | 3 | 4 |
| 14_ 7 Mal di testa dopo l’attività sessuale | 0 | 1 | 2 | 3 | 4 |
| 14_ 8 Infezione urogenitale | 0 | 1 | 2 | 3 | 4 |

15. Utilizzando la scala numerica riportata qui sotto, indichi quanto hanno influito sul Suo livello di attività sessuale i seguenti fattori durante l’ultimo mese. *(Una risposta è necessaria per ciascuna, anche se non Le è pertinente)*

|  | **0 *=*** Per niente | **1 *=*** Raramente, in meno del 25% delle occasioni | **2 *=*** Circa metà delle  occasioni | **3 *=*** Di solito, circa il 75% delle occasioni | **4 *=*** Sempre |
| --- | --- | --- | --- | --- | --- |
| 15_ 1 Problemi con la mia salute (p.e., infezioni, malattie) | 0 | 1 | 2 | 3 | 4 |
| 15_2 La salute del/la mio/a partner | 0 | 1 | 2 | 3 | 4 |
| 15_ 3 Conflitti nel nostro rapporto | 0 | 1 | 2 | 3 | 4 |
| 15_ 4 Mancanza di *privacy* | 0 | 1 | 2 | 3 | 4 |
| 15_ 5 Altro *(si prega di specificare)* | 0 | 1 | 2 | 3 | 4 |

16. Quanto è soddisfatto dell’aspetto complessivo del Suo corpo? *(Si prega di segnare la risposta più esatta)*

1. Molto soddisfatto
2. Abbastanza soddisfatto
3. Né soddisfatto né insoddisfatto
4. Abbastanza insoddisfatto
5. Molto insoddisfatto

17. Nell’ultimo mese, quanto frequentemente è riuscito a comunicare al/la partner i Suoi desideri o preferenze sessuali? *(Si prega di segnare la risposta più esatta)*

1. Non ho avuto un/a partner in questo periodo
2. Non sono riuscito a comunicare i miei desideri e preferenze
3. Raramente, in meno del 25% delle occasioni
4. Circa metà delle occasioni
5. Di solito, circa 75%
6. Sono sempre riuscito a comunicare i miei desideri e preferenze

18. Complessivamente, quanto è soddisfatto del rapporto sessuale col/la Suo/a partner? *(Si prega di segnare la risposta più esatta)*

1. Non ho avuto un/a partner in questo periodo
2. Molto soddisfatto
3. Abbastanza soddisfatto
4. Né soddisfatto né insoddisfatto
5. Abbastanza insoddisfatto
6. Molto insoddisfatto

19. Complessivamente, quanto crede che sia soddisfatto del Vostro rapporto sessuale il/la Suo/a partner? *(Si prega di segnare la risposta più esatta)*

1. Non ho avuto un/a partner in questo periodo
2. Molto soddisfatto
3. Abbastanza soddisfatto
4. Né soddisfatto né insoddisfatto
5. Abbastanza insoddisfatto
6. Molto insoddisfatto

20. Complessivamente, quanto Le importa nella vita la Sua attività sessuale? *(Si prega di segnare la risposta più esatta)*

1. Non è affatto importante
2. Non molto importante
3. Né importante né poco importante
4. Abbastanza importante
5. Molto importante

21. Indichi il numero che corrisponde alla frase che meglio descrive la Sua esperienza sessuale. (eterosessuale: che ha rapporti con persone dell’altro sesso; omosessuale: che ha rapporti con persone dello stesso sesso).

- - - 1. Interamente eterosessuale
      2. Principalmente eterosessuale, con qualche esperienza omosessuale
      3. Principalmente eterosessuale, ma con considerevole esperienza omosessuale
      4. Ugualmente eterosessuale e omosessuale
      5. Principalmente omosessuale, ma con considerevole esperienza eterosessuale
      6. Principalmente omosessuale, con qualche esperienza eterosessuale
      7. Completamente omosessuale

22. Indichi il numero che corrisponde alla frase che meglio descrive i Suoi desideri sessuali.

Interamente eterosessuale

Principalmente eterosessuale, con qualche desiderio omosessuale

Principalmente eterosessuale, ma con considerevole desiderio omosessuale

Ugualmente eterosessuale e omosessuale

Principalmente omosessuale, ma con considerevole desiderio eterosessuale

Principalmente omosessuale, con qualche desiderio eterosessuale

Completamente omosessuale

**S1.4. Translation of items different from those of the BISF-W**

| **Item** | **Italian** | **English** |
| --- | --- | --- |
| 14_0 | Eiaculazione raggiunta troppo presto | Ejaculation occurred prematurely |
| 14_ 2 | Difficoltà a raggiungere o mantenere l’erezione | Difficulty achieving or maintaining an erection |
| 14_4 | Eiaculazione non raggiunta o raggiunta con difficoltà | Ejaculation not reached or reached with difficulty |
| 14_8 | Infezione urogenitale | Urogenital infection |

**S1.5 BISF-M factors composition**

COMPUTE Dyadic = MEAN(Q10,Q5_7,Q11_7,Q7_5,Q7_7,Q9,Q5_5,Q13_4,Q13_3,Q17,Q13_2,Q5_1,Q7_1,Q12,Q11_5,Q4_4,Q4_6,Q7_6,Q5_6,Q13_1,Q7_4,

Q11_6, Q4_1,Q11_4,Q5_4,Q20,Q11_2) .

VARIABLE LABELS Dyadic 'Dyadic Sexuality’ .

EXECUTE .

COMPUTE Solitary = MEAN(Q4_2,Q7_3,Q5_3,Q11_3,Q5_2,Q3,Q7_2) .

VARIABLE LABELS Solitary 'Solitary Sexuality' .

EXECUTE .

COMPUTE Difficulties = MEAN(Q14_3,Q14_2,Q19,Q6,Q14_4,Q18,Q14_0,Q13_5) .

VARIABLE LABELS Difficulties 'Sexual Difficulties’ .

EXECUTE .

COMPUTE Anal = MEAN(Q5_8,Q7_8,Q11_8,Q4_7,Q4_5) .

VARIABLE LABELS Anal 'Anal Sexuality' .

EXECUTE .

**S1.6 Incoherent and Missing Scoring for the BISF-W**

COUNT

nmissF = Q1F Q2F Q3F Q4_1F Q4_2F Q4_3F Q4_4F Q4_5F Q4_6F Q4_7F Q5_1F Q5_2F Q5_3F Q5_4F

Q5_5F Q5_6F Q5_7F Q5_8F Q6F Q7_1F Q7_2F Q7_3F Q7_4F Q7_5F Q7_6F Q7_7F Q7_8F Q8F Q9F Q10F

Q11_1F Q11_2F Q11_3F Q11_4F Q11_5F Q11_6F Q11_7F Q11_8F Q12F Q13_1F Q13_2F Q13_3F Q13_4F

Q13_5F Q14_1F Q14_2F Q14_3F Q14_4F Q14_5F Q14_6F Q14_7F Q14_8F Q15_1F Q15_2F Q15_3F

Q15_4F Q15_5F Q16F Q17F Q18F Q19F Q20F Q21F Q22F (MISS) .

EXECUTE .

FREQUENCIES

VARIABLES=nmissF

/ORDER= ANALYSIS .

***coherence checking Q5F-Q7F-Q11F.

CROSSTABS

/TABLES=Q7_3F BY Q5_3F BY Q11_3F

/FORMAT= AVALUE TABLES

/CELLS= COUNT .

CROSSTABS

/TABLES=Q7_6F BY Q5_6F BY Q11_6F

/FORMAT= AVALUE TABLES

/CELLS= COUNT .

CROSSTABS

/TABLES=Q7_7F BY Q5_7F BY Q11_7F

/FORMAT= AVALUE TABLES

/CELLS= COUNT .

CROSSTABS

/TABLES=Q7_8F BY Q5_8F BY Q11_8F

/FORMAT= AVALUE TABLES

/CELLS= COUNT .

***Coherence filter construction.

COMPUTE coer_3F = 1 .

EXECUTE .

COMPUTE coer_6F = 1 .

EXECUTE .

COMPUTE coer_7F = 1 .

EXECUTE .

COMPUTE coer_8F = 1 .

EXECUTE .

COMPUTE coerF = 1 .

EXECUTE .

IF (Q5_3F = 0) & (Q7_3F>= 3) coer_3F = 0 .

EXECUTE .

IF (Q5_3F = 0) & (Q11_3F= 3) coer_3F = 0 .

EXECUTE .

IF (Q7_3F= 0) & (Q5_3F>= 3 ) coer_3F = 0 .

EXECUTE .

IF (Q7_3F = 0) & (Q11_3F >= 3) coer_3F = 0 .

EXECUTE .

IF (Q11_3F = 0) & (Q5_3F >= 3 ) coer_3F = 0 .

EXECUTE .

IF (Q11_3F = 0) & (Q7_3F >= 3) coer_3F = 0 .

EXECUTE .

IF (MISSING(Q5_3F) | MISSING(Q7_3F) | MISSING(Q11_3F)) coer_3F = 9 .

EXECUTE .

IF (Q5_6F = 0) & (Q7_6F >= 3 ) coer_6F = 0 .

EXECUTE .

IF (Q5_6F = 0) & (Q11_6F >= 3) coer_6F = 0 .

EXECUTE .

IF (Q7_6F = 0) & (Q5_6F >= 3 ) coer_6F = 0 .

EXECUTE .

IF (Q7_6F = 0) & (Q11_6F >= 3) coer_6F = 0 .

EXECUTE .

IF (Q11_6F = 0) & (Q5_6F >= 3 ) coer_6F = 0 .

EXECUTE .

IF (Q11_6F = 0) & (Q7_6F >= 3) coer_6F = 0 .

EXECUTE .

IF (MISSING(Q5_6F) | MISSING(Q7_6F) | MISSING(Q11_6F)) coer_6F = 9 .

EXECUTE .

IF (Q5_7F = 0) & (Q7_7F >= 3 ) coer_7F = 0 .

EXECUTE .

IF (Q5_7F = 0) & (Q11_7F >= 3) coer_7F = 0 .

EXECUTE .

IF (Q7_7F = 0) & (Q5_7F >= 3 ) coer_7F = 0 .

EXECUTE .

IF (Q7_7F = 0) & (Q11_7F >= 3) coer_7F = 0 .

EXECUTE .

IF (Q11_7F = 0) & (Q5_7F >= 3 ) coer_7F = 0 .

EXECUTE .

IF (Q11_7F = 0) & (Q7_7F >= 3) coer_7F = 0 .

EXECUTE .

IF (MISSING(Q5_7F) | MISSING(Q7_7F) | MISSING(Q11_7F)) coer_7F = 9 .

EXECUTE .

IF (Q5_8F = 0) & (Q7_8F >= 3 ) coer_8F = 0 .

EXECUTE .

IF (Q5_8F = 0) & (Q11_8F >= 3) coer_8F = 0 .

EXECUTE .

IF (Q7_8F = 0) & (Q5_8F >= 3 ) coer_8F = 0 .

EXECUTE .

IF (Q7_8F = 0) & (Q11_8F >= 3) coer_8F = 0 .

EXECUTE .

IF (Q11_8F = 0) & (Q5_8F >= 3 ) coer_8F = 0 .

EXECUTE .

IF (Q11_8F = 0) & (Q7_8F >= 3) coer_8F = 0 .

EXECUTE .

IF (MISSING(Q5_8F) | MISSING(Q7_8F) | MISSING(Q11_8F)) coer_8F = 9 .

EXECUTE .

IF (coer_3F=0 | coer_6F=0 | coer_7F=0 | coer_8F=0) coerF=0.

EXECUTE .

IF (coer_3F=9 | coer_6F=9 | coer_7F=9 | coer_8F=9) coerF = 9 .

EXECUTE .

FREQUENCIES

VARIABLES=coer_3F to coerF

/ORDER= ANALYSIS .

CROSSTABS

/TABLES=nmissF BY coerF

/FORMAT= AVALUE TABLES

/CELLS= COUNT

/COUNT ROUND CELL .

***eliminating incoherent answers in items q5, q7, q11 (putting missing).

***alternative 3.

DO IF (coer_3F = 0) .

RECODE

Q5_3F Q7_3F Q11_3F (ELSE=SYSMIS) .

END IF .

EXECUTE .

***alternative 6.

DO IF (coer_6F = 0) .

RECODE

Q5_6F Q7_6F Q11_6F (ELSE=SYSMIS) .

END IF .

EXECUTE .

***alternative 7.

DO IF (coer_7F = 0) .

RECODE

Q5_7F Q7_7F Q11_7F (ELSE=SYSMIS) .

END IF .

EXECUTE .

***alternative 8.

DO IF (coer_8F = 0) .

RECODE

Q5_8F Q7_8F Q11_8F (ELSE=SYSMIS) .

END IF .

EXECUTE .

***Recalculate missing.

COUNT

nmissF = Q1F Q2F Q3F Q4_1F Q4_2F Q4_3F Q4_4F Q4_5F Q4_6F Q4_7F Q5_1F Q5_2F Q5_3F Q5_4F

Q5_5F Q5_6F Q5_7F Q5_8F Q6F Q7_1F Q7_2F Q7_3F Q7_4F Q7_5F Q7_6F Q7_7F Q7_8F Q8F Q9F Q10F

Q11_1F Q11_2F Q11_3F Q11_4F Q11_5F Q11_6F Q11_7F Q11_8F Q12F Q13_1F Q13_2F Q13_3F Q13_4F

Q13_5F Q14_1F Q14_2F Q14_3F Q14_4F Q14_5F Q14_6F Q14_7F Q14_8F Q15_1F Q15_2F Q15_3F

Q15_4F Q15_5F Q16F Q17F Q18F Q19F Q20F Q21F Q22F (MISS) .

EXECUTE .

FREQUENCIES

VARIABLES=nmissF

/ORDER= ANALYSIS .

***Filter of subjects with more than 10 missing BISF-F.

USE ALL.

COMPUTE filter_$=(nmissF <= 10).

FILTER BY filter_$.

EXECUTE .

**S1.7 Incoherent and Missing Scoring for the BISF-M**

COUNT

nmissM = q2M q3M q4_1M q4_2M q4_3M q4_4M q4_5M q4_6M q4_7M q5_1M q5_2M q5_3M q5_4M

q5_5M q5_6M q5_7M q5_8M q6M q7_0M q7_1M q7_2M q7_3M q7_4M q7_5M q7_6M q7_7M q7_8M q8M q9M

q10M q11_0M q11_1M q11_2M q11_3M q11_4M q11_5M q11_6M q11_7M q11_8M q12M q13_1M q13_2M

q13_3M q13_4M q13_5M q14_0M q14_2M q14_3M q14_4M q14_6M q14_7M q14_8M

q15_1M q15_2M q15_3M q15_4M q15_5M q16M q17M q18M q19M q20M q21M q22M (MISSING) .

EXECUTE .

FREQUENCIES

VARIABLES=nmissM

/ORDER= ANALYSIS .

***coherence checking Q5M-Q7M-Q11M.

CROSSTABS

/TABLES=Q7_3M BY Q5_3M BY Q11_3M

/FORMAT= AVALUE TABLES

/CELLS= COUNT .

CROSSTABS

/TABLES=Q7_6M BY Q5_6M BY Q11_6M

/FORMAT= AVALUE TABLES

/CELLS= COUNT .

CROSSTABS

/TABLES=Q7_7M BY Q5_7M BY Q11_7M

/FORMAT= AVALUE TABLES

/CELLS= COUNT .

CROSSTABS

/TABLES=Q7_8M BY Q5_8M BY Q11_8M

/FORMAT= AVALUE TABLES

/CELLS= COUNT .

*** Coherence filter construction.

COMPUTE coer_3M = 1 .

EXECUTE .

COMPUTE coer_6M = 1 .

EXECUTE .

COMPUTE coer_7M = 1 .

EXECUTE .

COMPUTE coer_8M = 1 .

EXECUTE .

COMPUTE coerM = 1 .

EXECUTE .

IF (Q5_3M = 0) & (Q7_3M>= 3) coer_3M = 0 .

EXECUTE .

IF (Q5_3M = 0) & (Q11_3M= 3) coer_3M = 0 .

EXECUTE .

IF (Q7_3M= 0) & (Q5_3M>= 3 ) coer_3M = 0 .

EXECUTE .

IF (Q7_3M = 0) & (Q11_3M >= 3) coer_3M = 0 .

EXECUTE .

IF (Q11_3M = 0) & (Q5_3M >= 3 ) coer_3M = 0 .

EXECUTE .

IF (Q11_3M = 0) & (Q7_3M >= 3) coer_3M = 0 .

EXECUTE .

IF (MISSING(Q5_3M) | MISSING(Q7_3M) | MISSING(Q11_3M)) coer_3M = 9 .

EXECUTE .

IF (Q5_6M = 0) & (Q7_6M >= 3 ) coer_6M = 0 .

EXECUTE .

IF (Q5_6M = 0) & (Q11_6M >= 3) coer_6M = 0 .

EXECUTE .

IF (Q7_6M = 0) & (Q5_6M >= 3 ) coer_6M = 0 .

EXECUTE .

IF (Q7_6M = 0) & (Q11_6M >= 3) coer_6M = 0 .

EXECUTE .

IF (Q11_6M = 0) & (Q5_6M >= 3 ) coer_6M = 0 .

EXECUTE .

IF (Q11_6M = 0) & (Q7_6M >= 3) coer_6M = 0 .

EXECUTE .

IF (MISSING(Q5_6M) | MISSING(Q7_6M) | MISSING(Q11_6M)) coer_6M = 9 .

EXECUTE .

IF (Q5_7M = 0) & (Q7_7M >= 3 ) coer_7M = 0 .

EXECUTE .

IF (Q5_7M = 0) & (Q11_7M >= 3) coer_7M = 0 .

EXECUTE .

IF (Q7_7M = 0) & (Q5_7M >= 3 ) coer_7M = 0 .

EXECUTE .

IF (Q7_7M = 0) & (Q11_7M >= 3) coer_7M = 0 .

EXECUTE .

IF (Q11_7M = 0) & (Q5_7M >= 3 ) coer_7M = 0 .

EXECUTE .

IF (Q11_7M = 0) & (Q7_7M >= 3) coer_7M = 0 .

EXECUTE .

IF (MISSING(Q5_7M) | MISSING(Q7_7M) | MISSING(Q11_7M)) coer_7M = 9 .

EXECUTE .

IF (Q5_8M = 0) & (Q7_8M >= 3 ) coer_8M = 0 .

EXECUTE .

IF (Q5_8M = 0) & (Q11_8M >= 3) coer_8M = 0 .

EXECUTE .

IF (Q7_8M = 0) & (Q5_8M >= 3 ) coer_8M = 0 .

EXECUTE .

IF (Q7_8M = 0) & (Q11_8M >= 3) coer_8M = 0 .

EXECUTE .

IF (Q11_8M = 0) & (Q5_8M >= 3 ) coer_8M = 0 .

EXECUTE .

IF (Q11_8M = 0) & (Q7_8M >= 3) coer_8M = 0 .

EXECUTE .

IF (MISSING(Q5_8M) | MISSING(Q7_8M) | MISSING(Q11_8M)) coer_8M = 9 .

EXECUTE .

IF (coer_3M=0 | coer_6M=0 | coer_7M=0 | coer_8M=0) coerM=0.

EXECUTE .

IF (coer_3M=9 | coer_6M=9 | coer_7M=9 | coer_8M=9) coerM = 9 .

EXECUTE .

FREQUENCIES

VARIABLES=coer_3M to coerM

/ORDER= ANALYSIS .

CROSSTABS

/TABLES=nmissM BY coerM

/FORMAT= AVALUE TABLES

/CELLS= COUNT

/COUNT ROUND CELL .

*** eliminating incoherent answers in items q5, q7, q11 (putting missing).

***alternative 3.

DO IF (coer_3M = 0) .

RECODE

Q5_3M Q7_3M Q11_3M (ELSE=SYSMIS) .

END IF .

EXECUTE .

***alternative 6.

DO IF (coer_6M = 0) .

RECODE

Q5_6M Q7_6M Q11_6M (ELSE=SYSMIS) .

END IF .

EXECUTE .

***alternative 7.

DO IF (coer_7M = 0) .

RECODE

Q5_7M Q7_7M Q11_7M (ELSE=SYSMIS) .

END IF .

EXECUTE .

***alternative 8.

DO IF (coer_8M = 0) .

RECODE

Q5_8M Q7_8M Q11_8M (ELSE=SYSMIS) .

END IF .

EXECUTE .

***Recalculate missing.

COUNT

nmissM = q2M q3M q4_1M q4_2M q4_3M q4_4M q4_5M q4_6M q4_7M q5_1M q5_2M q5_3M q5_4M

q5_5M q5_6M q5_7M q5_8M q6M q7_0M q7_1M q7_2M q7_3M q7_4M q7_5M q7_6M q7_7M q7_8M q8M q9M

q10M q11_0M q11_1M q11_2M q11_3M q11_4M q11_5M q11_6M q11_7M q11_8M q12M q13_1M q13_2M

q13_3M q13_4M q13_5M q14_0M q14_2M q14_3M q14_4M q14_6M q14_7M q14_8M

q15_1M q15_2M q15_3M q15_4M q15_5M q16M q17M q18M q19M q20M q21M q22M (MISSING) .

EXECUTE .

FREQUENCIES

VARIABLES=nmissM

/ORDER= ANALYSIS .

*** Filter of subjects with more than 10 missing BISF-M.

USE ALL.

COMPUTE filter_$=(nmissM <= 10).

FILTER BY filter_$.

EXECUTE .

| **Table1S** |  |  |  |  |  |  |  |
| --- | --- | --- | --- | --- | --- | --- | --- |
| *Descriptive statistics (mean and standard deviation) of BISF factors by gender and age groups* | | | | | | |  |
|  |  | Women | | | Men | | |
|  |  | 18-29 years | 30-49 years | 50+ years | 18-29 years | 30-49 years | 50+ years |
|  |  | (n = 1925) | (n = 2763) | (n = 686) | (n = 717) | (n = 970) | (n = 493) |
| BISF | Dyadic | 3.00 (1.10) | 2.66 (1.06) | 2.05 (1.25) | 3.36 (1.06) | 3.28 (0.94) | 2.57 (1.11) |
|  | Solitaire | 1.93 (1.23) | 1.58 (1.21) | 1.01 (1.05) | 3.25 (1.26) | 2.78 (1.41) | 1.87 (1.33) |
|  | Difficulties | 1.18 (0.69) | 1.23 (0.66) | 1.19 (0.80) | 1.08 (0.55) | 1.12 (0.53) | 1.23 (0.64) |
|  | Anal | 0.44 (0.93) | 0.37 (0.85) | 0.25 (0.74) | 1.47 (1.68) | 1.46 (1.76) | 0.68 (1.26) |
